# Dynamic DNA cytosine methylation in the *Populus trichocarpa *genome: tissue-level variation and relationship to gene expression

**DOI:** 10.1186/1471-2164-13-27

**Published:** 2012-01-17

**Authors:** Kelly J Vining, Kyle R Pomraning, Larry J Wilhelm, Henry D Priest, Matteo Pellegrini, Todd C Mockler, Michael Freitag, Steven H Strauss

**Affiliations:** 1 Department of Forest Ecosystems and Society, Oregon State University, Corvallis, OR 97331, USA; 2 Molecular and Cellular Biology Program, Oregon State University, Corvallis, OR 97331, USA; 3 Dept. of Biochemistry and Biophysics, Oregon State University, Corvallis, OR 97331, USA; 4 Oregon Health Sciences University, Portland, OR 97002 USA; 5 Department of Botany and Plant Pathology, Oregon State University, Corvallis, OR 97331, USA; 6 Center for Genome Research and Biocomputing, Oregon State University, Corvallis, OR 97331, USA; 7 Department of Molecular, Cell and Developmental Biology, University of California, Los Angeles, Los Angeles, CA, 90024, USA; 8 The Donald Danforth Plant Science Center, St. Louis, MO 63132, USA

**Keywords:** Epigenetics, epigenomics, DNA methylation, 5-methylcytosine, *Populus*

## Abstract

**Background:**

DNA cytosine methylation is an epigenetic modification that has been implicated in many biological processes. However, large-scale epigenomic studies have been applied to very few plant species, and variability in methylation among specialized tissues and its relationship to gene expression is poorly understood.

**Results:**

We surveyed DNA methylation from seven distinct tissue types (vegetative bud, male inflorescence [catkin], female catkin, leaf, root, xylem, phloem) in the reference tree species black cottonwood (*Populus trichocarpa)*. Using 5-methyl-cytosine DNA immunoprecipitation followed by Illumina sequencing (MeDIP-seq), we mapped a total of 129,360,151 36- or 32-mer reads to the *P. trichocarpa *reference genome. We validated MeDIP-seq results by bisulfite sequencing, and compared methylation and gene expression using published microarray data. Qualitative DNA methylation differences among tissues were obvious on a chromosome scale. Methylated genes had lower expression than unmethylated genes, but genes with methylation in transcribed regions ("gene body methylation") had even lower expression than genes with promoter methylation. Promoter methylation was more frequent than gene body methylation in all tissues except male catkins. Male catkins differed in demethylation of particular transposable element categories, in level of gene body methylation, and in expression range of genes with methylated transcribed regions. Tissue-specific gene expression patterns were correlated with both gene body and promoter methylation.

**Conclusions:**

We found striking differences among tissues in methylation, which were apparent at the chromosomal scale and when genes and transposable elements were examined. In contrast to other studies in plants, gene body methylation had a more repressive effect on transcription than promoter methylation.

## Background

"Epigenetic" implies changes in regulatory states of genes or genomic DNA without changes in DNA sequence. The archetypical epigenetic modification in eukaryotic genomes is the addition of a methyl group to the fifth carbon of cytosine to produce 5-methylcytosine (5meC) [1,2], reviewed in [3]. Cytosine DNA methylation is an epigenetic modification that is shared by many eukaryotic organisms. Along with various other epigenetic modifications such as methylation, phosphorylation and acetylation of histone amino acids, cytosine methylation is an important regulator of biological processes including transposon silencing, heterochromatin organization, genomic imprinting, and gene expression.

The distribution of cytosine methylation is highly variable within plant genomes [4]. This overall methylation pattern, which is conserved among diverse plant taxa, is often described as "mosaic," as it consists of interspersed methylated and unmethylated regions [5,6]. The patterns of 5meC, mechanisms for *de novo *and maintenance methylation and the requirement for specific proteins for cytosine methylation have been best studied in *Arabidopsis thaliana*, where roughly 20% of the genome is methylated in whole seedlings [7,8]. Cytosine methylation is strongly enriched in heterochromatin at pericentromeric and subtelomeric repeats, and at rDNA clusters [7,9]. Repetitive sequences, which consist largely of transposons, retrotransposons, and tandem or inverted repeats, are highly methylated [5,10,11]. A novel and unexpected finding from genome-wide surveys was that a third of *A. thaliana *genes are methylated within their transcribed regions ("gene body methylation") [7,8], while perhaps 16% of rice (*Oryza sativa*) genes are enriched for 5meC [12]. The relationship between gene body methylation and transcription is currently not well understood. While promoter methylation is generally associated with lower transcription in *A. thaliana *[7], the relationship of gene body methylation to expression is complex, with methylation tending to occur most often in genes transcribed at moderate to high, but not very high, levels [4,7,8].

In plants, 5meC can occur in all sequence contexts (CG, CHG and CHH, where H refers to A, C or T) [13,14]. The mechanisms responsible for establishment and maintenance of 5meC are best studied in *A. thaliana *where the maintenance methyltransferase MET1 targets hemimethylated CG sites, and the *de novo *methyltransferases DRM2 and CMT3 target CHG and CHH sites. Disruption of maintenance methylation results in abnormal developmental phenotypes including stunting, malformed leaves, decreased apical dominance, lower fertility, disrupted heterochrony, delayed flowering time and abnormal flower morphology [15,16], while DRM2 and CMT3 mutants display defects in RNA mediated silencing [17], as well as dwarfing and abnormal leaf phenotypes [18]. The activity of methyltransferases appears synergistic, at least in some cases, so that deletion of DRM1/2/CMT3) affects CG methylation maintenance by MET1 [19]. Together, these results suggest that 5meC in all contexts can affect several aspects of chromatin regulation, with consequences for plant development and differentiation.

Tissue-level variation in methylation has been noted in several plant species. For example, in Arabidopsis, about six percent of cytosines were found to be methylated in immature floral [14], while 24 percent of CG, six point seven percent of CHG, and one point seven percent of CHH were methylated in young plants [20]. Few studies have compared high-resolution methylation profiles among tissues within a plant species. In rice, whole genome methylation patterns were found to be similar among mature leaves, embryos, seedling shoots and roots, but hypomethylation was correlated with preferential expression in endosperm [21]. Patterns of 5meC in LTR transposable elements differed between rice leaves and roots and affected transcription of neighboring genes [22] a phenomenon common to the SINE containing *FWA *promoter of *A. thaliana *[23,24].

In addition to well-established roles in transposable element silencing and genomic imprinting, DNA methylation may be involved in plant adaptation to stress [25,26]. In *A. thaliana*, genome-wide methylation increased in the progeny of plants exposed to temperature extremes, ultraviolet light [27], flood, and salt but decreased in progeny of drought-stressed plants [28,29]. In hybrid poplars (*P. deltoides *× *P. nigra*), shoot apices from drought-stressed juvenile trees exhibited genotype-dependent 5meC variation [30]. Differential DNA methylation patterns in poplar clones that have acquired differential transcriptome responses to drought stress have been observed [31].

While much has been learned from work on annual plants, in-depth investigations of cytosine methylation patterns in long-lived plants have been sparse. Because of their long term tissue differentiation and perennial exposure to environmental stresses, DNA methylation may play a greater role in both tree development and homeostasis. Studies of gross cellular DNA methylation indicate that it may vary substantially during tree development, whether assessed *in vivo *or *in vitro*. In apical buds of chestnut trees, *Castanea sativa*, 5meC increased during bud set and decreased during bud burst [32]. In Monterey pine, *Pinus radiata*, 5meC levels in needles of reproductively mature trees were double that of juvenile needles [33]. In shoots of chestnut and Monterey pine, a gradual increase in DNA methylation accompanied aging over 5-8 years [34,35]. Increased methylation in mature *vs*. juvenile leaves was associated with loss of capacity for *in vitro *organogenesis in *P. radiata *[36]. In micropropagated *Acacia*, shoots with juvenile leaves exhibited higher DNA methylation levels than shoots with mature leaves [37]. Transient DNA methylation of ovules accompanied embryogenesis in chestnut [38]. As noted above, in poplar drought stress induced changes in total cellular DNA methylation [30] and was associated with transcriptome changes within separately propagated clones [31].

A variety of experimental techniques can be applied to study genome-wide DNA methylation (reviewed in [39]). On a gross scale, the proportion of 5meC can be estimated by HPLC or HPCE, as has been done to show differences in 5meC among tissue types or treatments [33,35]. The drawback of these methods is the lack of sequence specific information. Immunoprecipitation with an antibody raised against 5-methylcytidine (MeDIP), followed by genome tiling array hybridization or high-throughput sequencing of the precipitated DNA (MeDIP-seq), has been used to enumerate and compare methylated regions in *Homo sapiens *[40,41], *Mus musculus *[42], *Neurospora *[43] and *A. thaliana *[7,8]. The most detailed, single-base resolution maps are generated by sequencing of genomic DNA treated with sodium bisulfite, which converts unmethylated cytosines to uracils but leaves 5meC unconverted [44]. However, this technique requires very high sequencing depth and is not suitable for mapping to repetitive genomic regions where uniqueness can be confounded by the presence of C to T SNPs. Genome-wide bisulfite sequencing was first used in Arabidopsis [14], but has now also been used to assess genome methylation in *Oryza sativa *and *P. trichocarpa *[6,21], as well as mammals including. *H. sapiens *[45] and *M. musculus *[46]. For the present work, we chose MeDIP-seq of many different tissue types because it provides comprehensive methylome coverage at a lower cost than genome-wide bisulfite sequencing.

The black cottonwood, *Populus trichocarpa*, is widely recognized as a reference species for tree biology. It has been studied in great detail over the past 30 years, and many resources are readily available, including a draft genome sequence http://www.phytozome.net/poplar, custom microarrays, and extensive transcriptome data [47]. For our studies we used genome assembly version 2.2 in combination with published expression microarray data from multiple tissue types [48,49]. While mature leaves from *P. trichocarpa *have recently been subjected to genome-wide bisulfite sequencing [50], high-resolution epigenomic methods have not yet been applied to discern tissue-level variation. We investigated variation in genome-level cytosine methylation among all of the major types of differentiated poplar tissues. To this end, we sequenced methylated DNA obtained by MeDIP from seven *P. trichocarpa *tissues on an Illumina GAIIx. We found overall patterns of cytosine methylation that are consistent with those seen in *Arabidopsis*, but observed differences in methylation patterns among tissue types not previously studied. We also found a different pattern of association of gene body methylation to gene expression.

## Results

### Collection of MeDIP-seq data

MeDIP-seq data representing three to five Illumina sequencing lanes were obtained for each of seven tissues (Table 1, Additional file 1). Each tissue sample consisted of two biological replicates, with the exception of xylem, for which there was a single biological replicate. "Pooled bud" data was compiled from separate MeDIP-seq tissue samples representing three bud dormancy stages (fall, winter, spring; a detailed study of dormancy-associated variation is in progress). Libraries prepared from non-immunoprecipitated "input" DNA from three biological replicates of fall bud tissue were sequenced as a control.

**Table 1:** Summary of MeDIP-seq experimental results.

Tissue	Biological Replicates	Platform	Lanes Sequenced	Total Reads (Illumina Yield)	No. Mapped Reads	Percent Mapped Reads
Spring bud	2	GAII	5	97,642,336	8,880,982	9.1
Autumn bud	1	GAII	3	27,397,024	10,836,547	39.6
Winter bud	1	GAII	3	26,301,421	9,734,237	37
Male catkin	2	GAII	5	78,702,151	24,870,095	31.6
Female catkin	2	GAII	5	63,861,351	17,212,810	27
Leaf	2	GA	4	70,264,008	13,524,682	19.2
Root	2	GA	5	81,017,743	9,284,891	11.5
Phloem	2	GAII	4	38,358,479	22,737,382	59.3
Xylem	1	GAII	5	33,649,277	12,278,525	36.5

		**Totals**	**39**	**517,193,790**	**129,360,151**	

Mapped reads include all non-clonal reads, allowing up to two mismatches.

### Validation of MeDIP-seq results by bisulfite sequencing

Bisulfite sequencing of eight selected targets was used to confirm quality of the MeDIP-seq data. Regions were selected to represent a range of RPKM values and maximum per-nucleotide coverage values (Additional files 2, 3), and were mainly at 5' ends of genes in promoters and coding regions. There was a strong correlation with both RPKM (R^2 ^= 0.92) and maximum per-nucleotide coverage (R^2 ^= 0.93) (Additional file 4). Cytosines in all three sequence contexts (CG, CHG, CHH) were methylated in the target regions, but in targets with an overall low cytosine percentage, the CHH context was more frequently methylated than CG or CHG (Additional file 5), and was more variable than the other contexts among the three examined bud stages (Additional file 6).

### Mapping of MeDIP-seq reads to the genome

Coverage of the total genome was calculated for each tissue type separately for uniquely mapping reads and for distributed repeats. Uniquely mapped non-clonal reads represented 9 to 59% of the total number of reads (Table 1). For non-immunoprecipitated control samples, uniquely mapping reads covered ~80% of the genome and k-mer redistributed repeats covered 23% of the genome; these percentages included overlaps at the ends of reads between the two types. In contrast, reads from MeDIP samples that were aligned to unique positions in the reference genome covered 26%-56% of the genome, while an additional 14%-19% of the genome was covered by distributed k-mer repeats (Additional file 7A). Within the covered portion of the genome, average coverage was deeper for distributed k-mer repeats (ranging from 4.9 reads/bp in xylem to 14.6 reads/bp in bud) than for uniquely mapping reads (ranging from 0.8 reads/bp in root to 2.5 reads/bp in bud) (Additional data file 7B).

**Figure 1:**
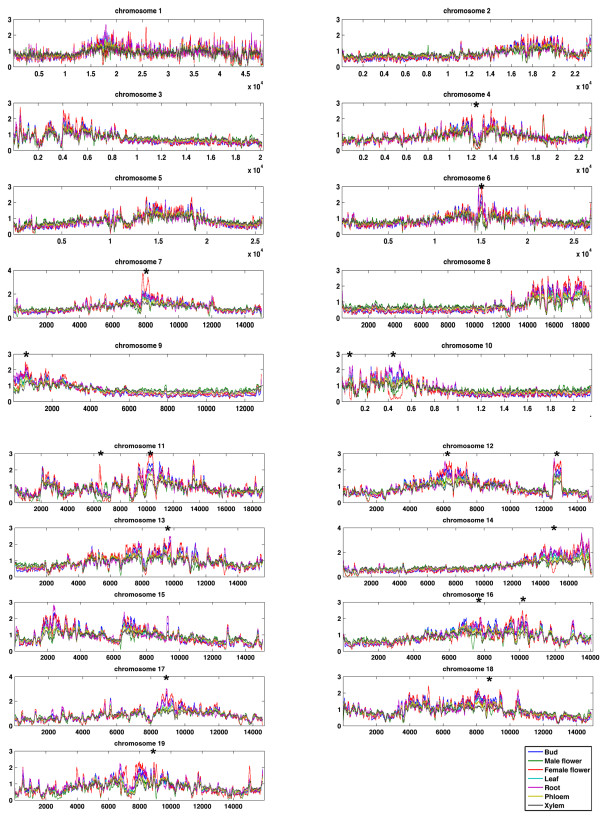
**Chromosome-level view of methylation among tissues**. A. Whole chromosome plots. MeDIP-seq reads were plotted in 1 kb windows along chromosomes. One line is shown for each tissue type. Asterisks indicate examples of large segments of high methylation variability among tissues.

**Figure 2:**
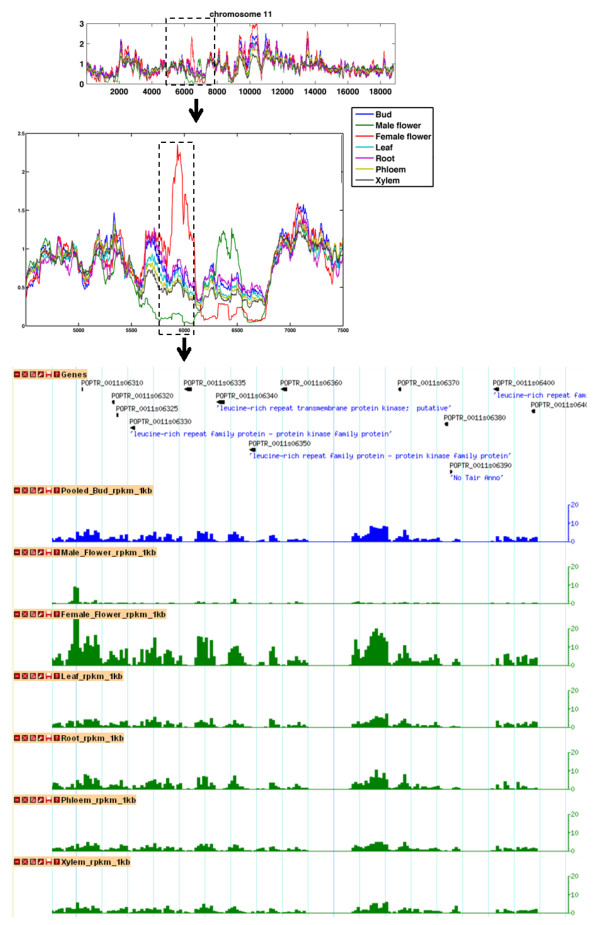
**Gene content in a region with methylation differences among tissues**. Zooming in on a region of chromosome 11 (dashed line) shows tissue-level variation at a locus containing a cluster of genes sharing the leucine-rich repeat (LRR) structural motif. Male and female catkins have a different methylation profile from other tissue types, and an apparent inverse pattern relative to each other over this region.

Fewer MeDIP-seq reads mapped to chromosomal regions where gene density was higher (Additional file 8). Several high-coverage regions also displayed high inter-tissue variability (Figures 1 and 2, Additional file 9). Eleven of the 19 chromosomes (I-IV, VI, VII, X, XI, XV, XVI, XIX) had high coverage by both unique reads and k-mer repeats that indicated possible centromeric or pericentromeric regions (Additional file 8). In all eleven cases, these regions corresponded to putative centromeres identified on the basis of high repeat-to-gene ratios that were also correlated with recombination valleys (P. Ranjan and G. Slavov, pers. comms.). In addition, our k-mer repeat maps correlated well with their equivalent "ambiguous reads" maps, for which no pre-selection process had been done prior to sequencing; this further supports our finding that genome regions with k-mer repeats tend to be more highly methylated than regions with uniquely mapped reads.

We identified methylation as statistically significant by applying three analytical methods to the 378,538 1-kb tiled windows that spanned the genome. The RPKM and CPPD methods, both at a 1% FDR, agreed in more than 60% of the windows called when assessing methylation within individual tissues; agreement was 92% and 49% when assessing windows that were ubiquitously methylated or unmethylated, respectively. Based on the windows in common among the two methods, 64% of the genome was unmethylated in all tissues and just over 2% was methylated in all tissues. As expected, the negative binomial analysis, when considered at p-value cutoffs of 10^-4 ^or 10^-3^, called many fewer methylated windows than the RPKM or CPPD methods. However, the windows that were called were mostly common to those called by the other two methods. At a p-value threshold of 10^-3^, 50% (methylated in all tissues) to 97% (methylated in leaf tissues) of the windows it called were in common with those called by both the RPKM or CPPD methods (Table 2).

**Table 2:** Number of methylated 1 kb windows called by three methods.

			No. methylated 1 kb windows			
	
All Tissues	RPKM		CPPD	NB	Common	Agreement NB
Methylated	13,715		14,036	2,335	1,096	46.9
Unmethylated	252,104		252,421	301,637	233,368	77.4
Tissue-specific	112,717		112,079	74,564	60,003	80.5
Tissue-specific proportion	0.89		0.89	0.97		

			**No. methylated 1 kb windows**			
	
**By Tissue**	**RPKM**		**CPPD**	**NB**	**Common**	**Agreement NB**

Bud	74,265		88,975	54,805	44,101	80.5
Male catkin	60,010		63,136	17,521	14,154	80.8
Female catkin	73,830		74,568	32,922	29,388	89.3
Leaf	66,951		63,207	26,858	20,937	97.5
Root	65,843		42,548	21,469	24,080	89.7
Phloem	61,792		71,652	6,499	4,838	74.4
Xylem	48,392		34,174	14,659	9,185	62.7

Number of 1 kb windows in each of seven tissues (bud, male catkin, female catkin, leaf, root, phloem, xylem) called methylated using RPKM cutoff (1% FDR), cumulative Poisson probability distribution (CPPD), or a negative binomial analysis (NB). "Common" refers to the number of 1 kb windows called methylated by all three of the analytic methods. Percent agreement NB refers to the percent of windows called by the NB method that were also in common with those called by both RPKM and CPPD. Tissue specific proportion is proportion of tissue-specific called windows as a fraction of all the windows in the genome that were found to be methylated in any tissues.

### Mapping of MeDIP-seq reads to genes

**Figure 3:**
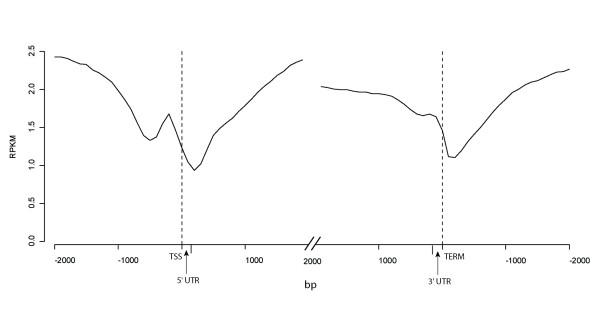
**DNA methylation within and proximal to gene models**. RPKM values of all genes with annotated UTRs were averaged over 100 bp tiled windows. Dashed lines delimit the transcribed region. The 5' and 3' UTRs shown represent the average size of these features in the *P. trichocarpa *genome. Windows were taken 2 kb upstream and downstream of the ends of 5' and 3' UTRs. Windows that overlapped adjacent gene models were excluded. If multiple splice variants of a gene model were annotated, only the first was used.

The *P. trichocarpa *v. 2.2 genome contains 39,756 annotated genes on chromosomal scaffolds. Of these, over all tissues, we identified 6,768 promoter-methylated genes (17.0% of all genes) and 6,207 body-methylated genes (15.6% of all genes), including genes that were methylated at both features. In order to determine patterns of 5meC relative to protein-coding genes, we used RPKM calculations to describe MeDIP-seq data distribution across promoters, 5' and 3' UTRs and coding regions (as well as introns and exons separately), and intergenic space. Gene promoters, gene bodies and intergenic regions had relatively high coverage in all tissue types from both unique reads and distributed repeats (Additional file 10). Across an idealized gene model, average RPKM values showed relatively high coverage in promoters, steadily decreasing 5' to 3', with a small peak 5' of the minimum at the transcription start site. There was higher coverage in the central portion of the transcribed region than at the 5' and 3' ends, and an increase of coverage 3' of the transcribed region (Figure 3).

### Variable methylation of transposable element classes

**Figure 4:**
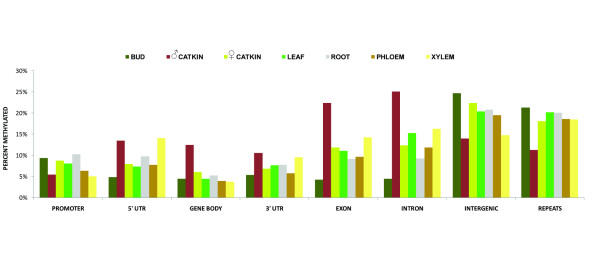
**Fraction of methylation among genome features and tissues**. Bars show the percent of each feature type determined to be significantly methylated (1% false discovery rate). As expected, intergenic and repetitive areas are most highly methylated. Gene body methylation - including exons and introns - is highest in male catkins.

**Figure 5:**
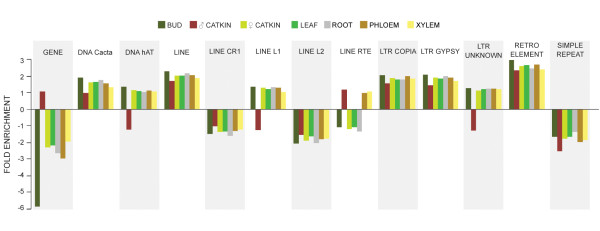
**Enrichment of repeat element methylation**. The frequency of elements from each category among the methylated regions of each sample is compared to the frequency of that class of element in the entire genome. Element genome coordinates and annotations were obtained from the RepPop database http://csbl.bmb.uga.edu/~ffzhou/RepPop.

When methylated regions were categorized by genome feature, intergenic (13.9-24.7%) and repetitive sequence features (11.2-21.3%) were the most frequently methylated (Figure 4). Counts of methylated genes and transposable elements were compared to each group's overall frequency in the genome, and genes, short repeats, and two subcategories of LINE repeat elements were underrepresented among methylated regions, while retroelements, LTR transposons, hAT and Cacta elements, and two other subcategories of LINEs were enriched (Figure 5). Methylation in male catkins was an exception to the overall trend, with genes overrepresented, and hAT, LINE1 and unknown LTR elements underrepresented.

### Differentiation in methylation among tissues

On a chromosome scale, overall MeDIP-seq read coverage was similar across tissue types, but there were visually striking regions of large-scale heterogeneity among tissues that ranged from approximately 100 kb to 2 Mb in length (Figures 1 and 2, Additional file 9; several examples are indicated with asterisks in Figure 1). Many of these areas of methylation heterogeneity had low gene density and contained clusters of transposable elements, but one region we examined more closely was a cluster of leucine-rich repeat (LRR) genes. The regions with the highest methylation tended to show the highest tissue-associated variation in methylation (Additional file 11). However, there were also large chromosomal sections where methylation signals were relatively low for all tissues, but a particular tissue was consistently highest (e.g., the right half of chromosome 3 and the left half of chromosome 8 in Figure 1).

**Figure 6:**
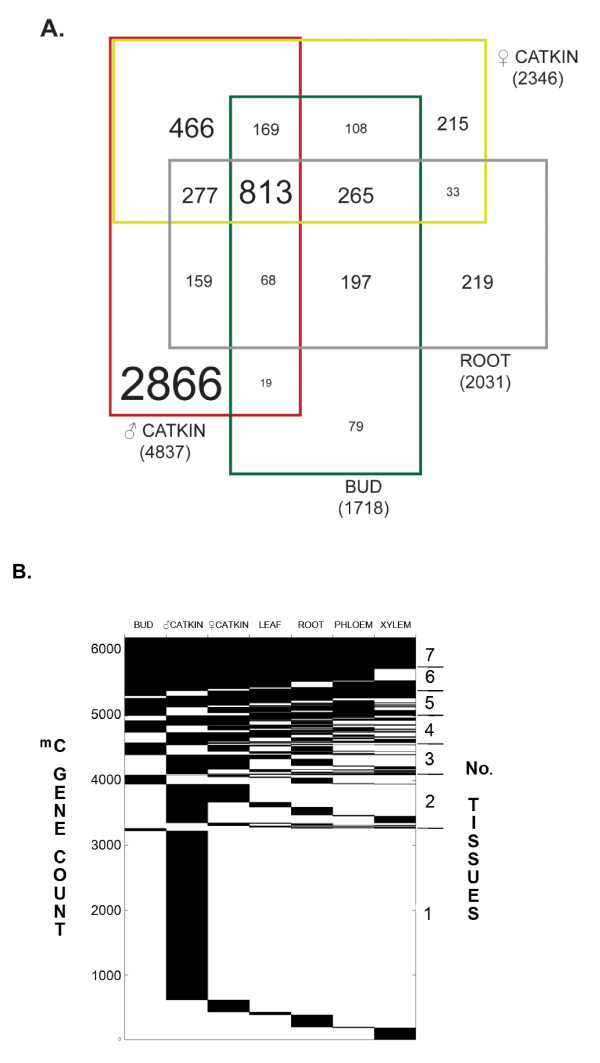
**Differentiation of gene body methylation among selected tissues**. A. Venn diagram showing overlaps of gene body methylation among four of the sampled tissue types. Numbers are counts of genes called methylated (RPKM compared to non-immunoprecipitated input, 1% false discovery rate). B. Presence/absence heat map showing blocks of body- methylated genes common (black) among the seven sampled tissues. Left y-axis shows counts of genes.

**Figure 7:**
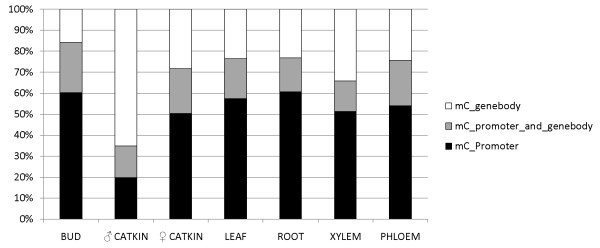
**Frequency of gene body vs. promoter methylation among tissues**. Among genes with promoter and/or gene body methylation, promoter methylation is more prevalent, and both features are methylated ~15-30% of the time. The exception is male catkin, for which gene body methylation is prevalent.

Genome-wide methylation in different tissues, determined using 1-kb tiled windows across the genome as described above, showed that 33.7% of the genome was differentially methylated. Further, pairwise tissue methylation comparisons based on the 1-kb windows showed substantial differential methylation (Table 3), with an overall mean pairwise similarity of 31.5%. Male catkins had by far the greatest number of gene-body-methylated genes that were not methylated in any other tissue type (2,866) (Figure 6). Seventeen to 31% of gene models methylated in any tissue had both promoter and body methylation (Figure 7). Within a tissue type, promoter-methylated genes were more frequent than body-methylated genes, accounting for 50-60% of all methylated genes. Roots accounted for 41% of promoter-methylated genes that were restricted to one tissue type, and male catkins accounted for 80% of single-tissue body-methylated genes. When gene-associated features were compared among tissues, there was also extensive tissue-associated variation (Table 4). Promoters methylated in common among tissues ranged from a maximum of 16% (leaf vs. root) to less than one percent (male catkin vs. phloem, root, or bud). Gene bodies methylated in common ranged from 11% (root vs. phloem) to less than one percent (male catkin vs. bud or female catkin vs. leaf, root, xylem, of phloem).

**Table 3:** Statistical comparision of called methylated 1 kb windows between tissues.

	**Male catkin**	**Female catkin**	**Leaf**	**Root**	**Xylem**	**Phloem**	**Bud**
	
**Male catkin**	**63,136**	0.27	0.32	0.53	0.51	0.32	0.32
	
**Fem. catkin**	0.50	**74,568**	0.39	0.49	0.62	0.42	0.23
	
**Leaf**	0.33	0.18	**63,207**	0.36	0.42	0.13	0.12
	
**Root**	0.30	0.17	0.20	**42,548**	0.44	0.23	0.07
	
**Xylem**	0.19	0.13	0.14	0.34	**34,174**	0.05	0.11
	
**Phloem**	0.31	0.17	0.17	0.41	0.32	**71,652**	0.12
	
**Bud**	0.59	0.30	0.41	0.44	0.67	0.48	**88,975**

Differences among tissues were examined by generating all possible pairwise comparisons in 1 kb read-count windows using the CPPD method. Each entry is the ratio of the number of differentially methylated windows in the comparison (row tissue compared to column tissue) divided by the sum of the union of the methylated windows in either tissue compared to input. The total number of methylated windows in each tissue is shown on the diagonal.

**Table 4:** Statistical comparison of called methylated genic features between tissues.

	**Male catkin**	**Female catkin**	**Leaf**	**Root**	**Xylem**	**Phloem**	**Bud**
	
**Male catkin**	**2,095/4,837**	0.028	0.013	0.006	0.021	0.0081	0.0066
	
**Female catkin**	0.070	**3,397/2,346**	0.018	0.024	0.013	0.014	0.069
	
**Leaf**	0.021	0.0049	**3,106/1,726**	0.16	0.036	0.13	0.079
	
**Root**	0.023	0.0075	0.050	**3,965/2,031**	0.029	0.11	0.094
	
**Xylem**	0.026	0.0045	0.028	0.031	**1,940/1,431**	0.10	0.048
	
**Phloem**	0.011	0.0039	0.084	0.11	0.071	**2,473/1,505**	0.065
	
**Bud**	0.0043	0.037	0.035	0.053	0.023	0.031	**3,642/1,718**

Sets of significantly methylated gene-associated features compared among tissues determined by the RPKM method. Gene counts per tissue are shown on the diagonal as: number with significantly methylated promoter/number with significantly methylated gene body. Proportions of these sets shared by pairs of tissues is shown, with promoter commonalities above the diagonal and gene body commonalities below the diagonal.

### Gene ontology of methylated genes in male catkins

To determine the functional classification of body-methylated genes specific to male catkins, we tested for enrichment of gene ontology (GO) categories. This analysis revealed significant enrichment (p < 0.05) in 168 specific gene ontology categories, including those related to translation/protein metabolism (264 genes), nucleic acid binding (322 genes) and RNA metabolism (135 genes). Some of the enriched GO categories observed are illustrated in Additional file 12.

### Association of methylation and gene expression

We compared the categorized gene feature methylation to tissue-specific gene expression data from previous expression microarray studies [48]. We did this on both a global scale, looking at methylation and expression data pooled across all tissue types, and on a per-tissue basis. We also analyzed the association of gene expression to methylation at particular genic features. Clustering of tissues by only their overall gene expression patterns suggested that floral tissues, the bud samples, and root and xylem had the most similar gene expression profiles (Additional file 13). However, when tissues were clustered based on only RPKM data, the patterns were highly dissimilar (Additional file 14). The biological replications clustered for both the male and female inflorescence tissues, as well as for the buds and input samples. However, the biological replications for the root, leaf and phloem tissues did not cluster, and the positions of all tissues bore little similarity to what was observed based on gene expression data. The lack of concordance was also observed when biological replications were pooled and methylation of gene bodies or promoters clustered (data not shown). Thus, at the gross genome level, tissue specific gene expression and methylation had no obvious association.

**Figure 8:**
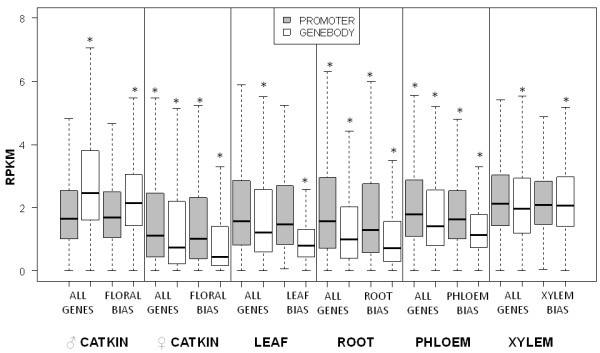
**Association of tissue predominant expression with methylation**. RPKM data from lists of genes with strong bias in gene expression compared to those in the entire genome for those tissues. Asterisks indicate significantly different means (P < 0.05) based on comparisons of "all genes" to "tissue bias" genes within a tissue type group, calculated for promoters and gene bodies separately.

To further test the hypothesis that gene methylation differences among tissues were correlated with tissue predominant gene expression, we interrogated our methylation data using lists of genes determined to have high tissue-predominant ("biased") expression based on calculations in Rodgers-Melnick et al. [49]. They used much of the same microarray dataset as analyzed in this paper to identify sets of genes with high levels of tissue differential expression by applying the formula:

$$Bias=\frac{\left(w_{s}*E_{s}\right)}{\left(w_{s}*E_{s}\right)+\left(w_{o}*E_{o}\right)}$$

for which tissues were divided into subsets *s *and *o*, with n_s _assigned to the number of tissues in subset *s*, and n_o _was assigned to the number of tissues not in subset *s *(all other tissues); w_s _denotes the weight applied to tissues in subset s, i.e. max(n_s_, n_o_)/n_s_, w_o _denotes the weight applied to tissues in subset o, i.e. max(n_s_, n_o_)/n_o_, E_s _denotes the sum of expression values of tissues in subset s, and E_o _denotes the sum of expression values of tissues in subset. A gene was considered biased at or above a calculated bias of 0.9. The number of tissue biased genes varied from 320 for leaves to 6,729 for male and female catkins (pooled for the calculation of expression bias). From this set, we found that 2.5 to 5.8% and 0.5 to 12.5% of genes called as biased based on expression were called methylated by our criteria at promoters or gene bodies, respectively. When all of the genes showing bias for a tissue type were compared to all genes in our dataset for their RPKM levels, the differences were small; however, 9 of 12 comparisons were statistically significant and consistent in direction (Figure 8). In all cases where a difference was significant, the tissue predominant genes had lower methylation, both for promoters and/or gene bodies. In every case, whether significant or not, the genes with upwardly biased expression in that tissue never showed a higher RPKM value for promoters or for gene bodies. Excluding male catkins as outliers due to their unusual GO patterns (discussed above), for gene bodies all six tissues were consistent in having lower RPKM for the expression-biased tissue set (P < 0.05).

**Figure 9:**
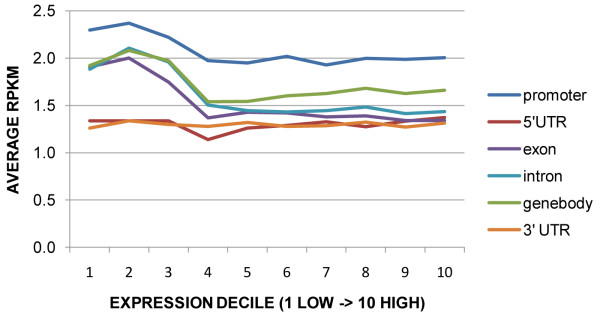
**Gene expression in relation to methylation at genic features**. The genes with lowest expression tend to have higher methylation at introns, exons, promoters and gene bodies (which include exons and introns), but not at UTRs. Gene models were binned in deciles representing low to high expression levels based on Nimblegen microarray data. RPKM data was pooled across all tissue types.

**Figure 10:**
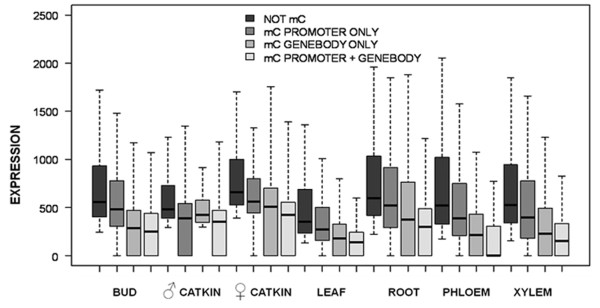
**Gene expression in relation to promoter and gene body methylation**. Box plot showing average expression of unmethylated genes, genes with methylated promoters, and genes with methylated gene bodies was compared for each tissue type using gene expression data from a Nimblegen microarray. Each box encloses the middle 50% of the distribution (25^th ^percentile - 75^th ^percentile, or interquartile range (IQR)). Lines in boxes mark medians. Lines extending from boxes mark minimum and maximum values that fall within 1.5 times the IQR.

**Figure 11:**
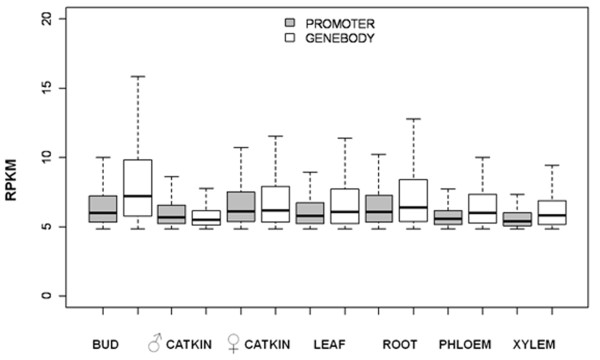
**Methylation in gene bodies vs. promoters for significantly methylated genes**. Data shown is for genes with methylation at promoters only or gene bodies only for genes called methylated (exceeding 1% false discovery rate). A similar result was obtained when genes with methylation at both features are included.

When MeDIP-seq reads and transcript abundance were pooled across tissue types, a much stronger association of methylation and gene expression was evident (Figure 9). The lowest three deciles of expressed genes had significantly higher RPKM values (p < 0.05) for both promoters and gene bodies than genes in higher expression deciles. The 5' and 3' UTRs, however, were unassociated with gene expression. The pattern of increased methylation in promoters and/or gene bodies for the most weakly expressed genes was also consistent (p < 0.05) when gene expression was examined by tissue type. All seven tissues had consistent patterns when the top three and bottom seven deciles were considered for gene body, exon and intron: The lowest three deciles of expressed genes had higher mean RPKM values and a much wider RPKM range. This trend was also present for promoters in all tissues except for male catkins. As expected, genes with the highest expression were called unmethylated, whereas methylation at genes and/or promoters was associated with reduced expression (Figure 10). In all seven tissues, non-methylated genes had higher expression than the other three categories of genes shown, and those with only methylated gene bodies had higher expression than those with both methylated gene bodies and promoters (P < 0.05). Male catkins were again somewhat of an exception to otherwise highly consistent patterns; genes with only body methylation had a narrower range and slightly higher median expression than promoter-methylated or promoter-and-body-methylated genes. Among the subset of significantly methylated genes, with male catkins again excluded as an outlier, gene body methylation was significantly higher than promoter methylation in all tissues (P < 0.05) (Figure 11).

## Discussion

We have taken advantage of two of poplar's characteristics as a model tree species--high quality genomic resources and extensive, highly elaborated tissue types--to interrogate epigenomic variation at genome scale. Previous studies have either compared total DNA methylation among different tissue types, or have looked at one or very few tissues at genomic scale. Most high-resolution studies of methylation in *Arabidopsis *and rice have used seedlings or young plants, which are complex mixtures of different tissues rather than discrete tissue types, each composed of their own complex cell types. Only a handful of previous studies have examined genome-wide, high-resolution methylation differences among coherent tissue types [6], and these have mainly focused on comparisons of cytosine methylation in endosperm and embryo during seed development [51].

The MeDIP-seq method does not provide single-base resolution as does genome scale bisulfite sequencing, and therefore does not allow detailed analysis of cytosine context in methylated regions. However, region-specific bisulfite sequencing validated our MeDIP-seq results, showing that both calculated RPKM and maximum coverage per nucleotide reflect the underlying percentage of methylated cytosines in regions with varying cytosine content and position relative to genes. Within bisulfite-sequencing target regions, cytosines in all three (CG, CHG, CHH) sequence contexts were identified, with CG and CHG methylation being more consistent within tissues. However, in the two targets with cytosine content < 10%, cytosines in CHH context were methylated more frequently than those in the other two contexts. One of these targets was 5' of a gene model, and the other spanned the 5' end of a gene model coding region. Previous studies have reported that CGs are more frequently methylated than CHGs or CHHs, especially in coding regions, and 5meCHH, while less frequent in general, is more common in repeat regions and short transposable elements [6,11,20].

### Differential tissue methylation was extensive at genic and non-genic regions

MeDIP-seq read calculations and RPKM and CPPD statistical analysis based on differences among 1-kb genome windows showed that only ~2% of the *P. trichocarpa *genome was methylated in all seven assayed tissues. In contrast, 64% of the genome was ubiquitously unmethylated. The difference implies that one-third of the genome was differentially methylated among the tissues studied. Comparisons of promoter- and gene body methylated genes likewise showed extensive tissue differential methylation; 11 to 16% or less were methylated in common among tissues. We know of no comparable estimates of tissue-level variation in other plants; the few studies that have compared gene-level variation in different tissue types have used small numbers of tissues and reported low 5meC variation among tissues, most of which were accounted for by variation in transposable element methylation [21]. At least some of the chromosome blocks we observed that had highly tissue-differentiated methylation were also rich in transposable elements (Additional file 9).

### Chromosome methylation supports the locations of putative centromeres

We separately mapped unique MeDIP-seq reads and k-mer repeat reads, distributing k-mer repeats over all their genome occurrences. The *P. trichocarpa *genome is highly duplicated, with ~41% of the assembled genome considered repetitive (based on 16-mer counts > 34; [52]). Due to the difficulty of assembling repetitive genome regions, k-mer repeats were masked from the original genome assembly [47]. This repeat exclusion may be the reason that unique reads covered a much larger proportion of genome space than k-mer repeat reads. On a chromosomal scale, repeat regions were also correlated with genome gaps; the v2.2 assembly includes a large number (2,499) of scaffolds that are not yet assigned to specific chromosomes.

Our chromosome methylation maps showed concentrations of MeDIP-seq reads, in particular k-mer repeats, on more than half of *P. trichocarpa *chromosomes. These regions correspond with areas of low gene density, which are expected for centromere/pericentromere locations. A similar chromosome methylation profile, with high methylation in centromeric and pericentromeric regions, has been observed in *Arabidopsis *[7,51]. Centromeric satellite repeats are generally methylated and silenced [53], although repeats associated with centromere-specific histone CENH3 are hypomethylated compared to their counterparts in pericentromeric heterochromatin [54,55]. Genes near centromeres are also likely to be methylated [8]. Chromosomes lacking a single methylation peak had either more than one distinct methylation peak (e.g. LG V, LG XII), or more broad, indistinct methylated regions (e.g. LG XIII, LG XVII). These regions likely reflect the large chromosomal rearrangements and segmental duplications that mark the evolutionary history of *Populus *[47].

### Retroelements showed extensive and differential tissue methylation

Our data showed that protein-coding genes were underrepresented in the methylated fraction of the genome, while transposable elements and other simple repeats were generally methylated. LTR-gypsy retroelements are abundant in heterochromatic centromeric and pericentromeric regions in plants, and are the most plentiful repetitive element in the *P. trichocarpa *genome [52]. We found that this retroelement class was also enriched in the methylated fraction of the genome in all tissue types. Four other retroelement categories (DNA cacta, LINE, LTR copia, retroelement) were also overrepresented in the methylated genome fraction, which is not surprising given the extensive evidence of methylation-mediated transposable element silencing in eukaryotic genomes [56,57]. Two classes of LINE elements (LINE CR1, LINE L2) were underrepresented among the methylated genome fraction, and one class of LINE elements (LINE LTE) was overrepresented in xylem, phloem and male catkins, but underrepresented in buds, female catkins, leaves and roots. Thus, these elements showed considerable differential methylation by tissue. LINE elements are more abundant in *Populus *compared to other plant genomes, and there appears to have been a recent expansion of this element class in the genome [52].

### Genes were extensively methylated

Four to 12% of annotated protein-coding genes were methylated, with the level varying widely among tissues as discussed above. This is lower than the estimated 30% of methylated transcribed regions in *Arabidopsis *[7], but closer to the 16% predicted for rice [12]. The pattern of methylation within and around protein-coding genes was consistent with that seen in previous studies [6,14,20,50], with methylation high 5'and 3' of the transcribed portion of genes. Within the transcribed region, methylation was lowest near the transcription start and stop sites and increased away from there within the gene body. Interestingly, we observed a prominent methylation peak ~200 bp 5' of the transcription start site. A similar peak was seen in methylation profiles of *A. thaliana *embryos and endosperm in one study [51], but not in a second study [58]. In *Oryza *spp., a small 5' peak was seen for methylation in CHH context, but not CHG or CG context [6], while no spike in methylation in any sequence context in this region was identified elsewhere [50]. The cause for both the apparent peak and the incongruity of results remains unclear.

### Promoter and gene body methylation is negatively correlated with transcription

Our data showed that promoter-methylated genes had a wider expression range and higher median expression than body-methylated genes in most tissues. Methylation upstream or downstream of genes is generally understood to repress transcription [7,8,59]. Our results support this notion, as promoter-methylated genes had lower expression than genes that were not called methylated at any feature. Surprisingly, our results also indicated that gene body methylation was more repressive of transcription than promoter methylation. This contradicts what has been reported for *Arabidopsis*, where body-methylated genes are often highly transcriptionally active [7,59]. However, the relationship between gene body methylation and gene expression in plants appears to be confounded by additional factors such as gene length [8], and additional local epigenetic modifications. DNA methylation in gene bodies may not cause either absence or presence of transcription at all but rather mark splice junctions and thus be correlated to gene expression [8].

Several studies have examined the transcriptional effects of combinations of 5meC and histone modifications: In *Arabidopsis *seedlings, histone 3 lysine 4 monomethylation (H3K4meI) was highly correlated with CG-context methylation in transcribed regions of transcriptionally-active genes [60], while H3K27me3 was anticorrelated [61]. In *Zea mays *roots and shoots, genes with low levels of transcription had either 5meC or H3K27me3, also in an apparent mutually exclusive pattern [62]. In rice shoots, a complex pattern was observed, with hypermethylated genes tending to have fewer histone modifications and lower transcription, while hypomethylated genes exhibited a range of expression, with concurrent H3K4me3 associated with higher transcription levels, and concurrent H3K27me3 associated with lower transcription levels [12]. The emerging picture is of a complex hierarchy of combinations of 5meC with other epigenetic modifications, in addition to overall sequence context and chromatin context, that ultimately regulate transcription.

We examined the correlation between methylation and tissue predominance of gene expression in two ways: by comparing hierarchical clustering patterns of gene methylation and expression, and by querying methylation status of sets of genes deemed to be expressed in a tissue-preferential manner. Hierarchical clustering patterns revealed no large scale, consistent tissue-level patterns between methylation and expression. In *Zea mays*, DNA methylation in shoots and roots was also uncorrelated with differential gene expression on a genome scale [62]. However, when methylation profiles of sets of genes with tissue-biased expression were examined, they did show differences in promoter and gene body methylation. Though small on average, the differences were highly statistically significant and consistent between promoters and gene bodies. This analysis suggests that DNA methylation may indeed play a role in directing or maintaining tissue differential gene expression, though its extent appears modest. To our knowledge, this is the first observation of genome scale tissue differentiation of gene expression with DNA methylation in plants.

### Male catkins showed a unique pattern of methylation and associated gene expression

Surprisingly, male catkins had a far greater number of genes with body methylation than other sampled tissues, and the level of methylation of these genes was lower than that observed in other tissues. Expression of gene body-methylated genes was also higher than in other tissues except for female catkins. Three retroelement categories (DNA hAT, LINE1, LTR unknown) were underrepresented in the methylated fraction in male catkins, but overrepresented in all other tissue types. These unusual patterns seen may reflect the demethylation and reactivation of several types of transposable elements in pollen vegetative nuclei, with the associated siRNA cascade silencing transposable elements in sperm nuclei. Our male catkins were collected at anthesis (pollen release) and the majority of their biomass appeared to be made up of dehiscing (drying and opening) anthers; pollen DNA can therefore be expected to be highly represented in our male catkin data. Perhaps hypermethylation of surrounding transposable elements could also result in some associated low-level methylation of protein-coding genes, resulting in the unusual pattern of genic methylation seen. Genes that were body-methylated only in male catkins and not in the other tissue types had lower expression in male catkins than in all other tissues types except leaves, and gene ontology categorization of these genes showed enrichment of categories related to protein metabolism, cellular signaling, and DNA/RNA binding. At least some of these genes may play a role in pollen-associated changes in small RNA metabolism and associated DNA methylation. In contrast, female catkins did not show a distinctive pattern of DNA methylation or associated gene expression, even though genome-wide demethylation has been observed in endosperm relative to embryo tissue [58]. Active demethylation is brought about by DEMETER, which is expressed specifically in the central cell of the female gametophye and removes methylated cytosines via a mechanism involving single-strand break repair [63,64]. We believe the difference between male and female catkins was mainly because we collected female catkins during early pollen release, well before endosperm and embryo development was likely to have begun on a large scale. In addition, examination of a subset of our collected female inflorescences did not show any signs of seed development when a subsample of ovules was dissected (data not shown).

## Conclusions

Epigenomic studies have been applied to very few plant species to date. Our study is the first description of epigenomic differentiation among tissues in in any tree or perennial plant species at genome scale resolution. We sequenced methylated DNA from seven distinct tissues representing a wide range of developmental variation. Although the general pattern of chromosome and genic methylation agree with those of Arabidopsis and rice, there were a number of important differences or elaborations that may relate to its distinctive biology and evolution, and warrant further analysis. These include the degree of tissue-specific methylation throughout the genome and its association with genes; the negative association of gene body methylation with gene expression; the modest but consistent association of tissue-differential gene expression with promoter and gene body methylation; the peak in methylation 5' to genes; and the distinctive pattern of male catkin transposon and gene body methylation. The genomic catalog provided will also provide a foundation to inform a variety of other investigations, including those related to natural variation in rate of recombination throughout the genome, position effects observed during genetic engineering, and the interspecific heterosis and gender differentiation (dioecy) that are observed in poplar and many other plant species.

## Methods

### Plant material

Genomic DNA for most tissues was obtained from *P. trichocarpa *clone Nisqually-1, the genotype that was used for the published genome sequence [47]. Mature leaves were collected in September 2008, and buds were collected in August-September 2008, December 2008 and March 2009 from two-year-old trees at a field site in Corvallis, Oregon, USA. Fine roots and xylem and phloem ~15 cm below the apical bud were collected in August 2009 from two-year-old Nisqually trees maintained in a lath house at Oregon State University, Corvallis, Oregon. Male and female catkins were collected at anthesis in March, 2009 from mature wild *P. trichocarpa *in Corvallis, Oregon. Male catkins were collected at the start of pollen shed, and female stigmas had adhering pollen, but dissection of a small sample (~20) ovules from several different inflorescences showed no signs of seed development.

### Methylated DNA immunoprecipitation

The DNA extraction method was based on a previously published method [65]. Approximately 250 mg of tissue was ground to a fine powder in liquid nitrogen, then homogenized in extraction buffer (1 ml; 50 mM Tris [pH 8], 5 mM EDTA, 0.35 M sorbitol, 10% [w/v] polyethylene glycol [MW 8000], 1% [w/v] N-laurylsarcosine, 0.1% [w/v] bovine serum albumin (BSA), 0.1% [v/v] β-mercaptoethanol, 1% hexadecyltrimethylammonium bromide, 2 M NaCl). The homogenate was incubated at 60-65°C for 60 min in sterile 1.5 ml microcentrifuge tubes, followed by extraction with ~500 μl of 24:1 phenol:chloroform. Following centrifugation at 13,000 × g for 10 min, the aqueous layer (200-300 μl) was moved to a new, sterile 1.5 ml microfuge tube. DNA was precipitated with two volumes of ice-cold 95% ethanol at 4°C for 2-24 hours and subsequently pelleted by centrifugation at 13,000 × g for 5 min. The pellet was rinsed with 500 μl of 70% ethanol, then dried in a speed-vac for 5 min and finally resuspended in 50 μl TE buffer. Fifty microliters of 10 μg/ml RNase enzyme (Qiagen, Valencia, CA) were added and the mixture incubated at 37°C for 60 min to digest RNA. DNA concentration was determined using an ND-1000 spectrophotometer (Thermo Fisher Scientific, Waltham, MA).

Prior to immunoprecipitation, genomic DNA was sheared to 200-1000 bp fragments and ligated to Illumina sequencing adaptors as described previously [66]. Ten to twenty micrograms of genomic DNA were diluted to 300 μl in TE buffer. The DNA was sheared for 18 min with 30 sec on/off cycling at 4°C in a Diagenode Bioruptor (Sparta, NJ). The sheared fragments were recovered by using a Qiaquick PCR purification kit (Qiagen) according to manufacturer's instructions (final elution volume 52 μl). The fragments were end-repaired by mixing 50 μl of the DNA sample, 25 μl sterile distilled H_2_O, 10 μl T4 DNA ligase buffer (Invitrogen, Carlsbad, CA), 4 μl 20 mM dNTP mix, 5 μl T4 DNA polymerase (Invitrogen or New England Biolabs, Ipswich, MA), 1 μl Klenow DNA polymerase (Invitrogen or New England Biolabs) and 5 μl T4 polynucleotide kinase (New England Biolabs) incubated for 30 min at room temperature (San Diego, CA) were ligated to the DNA after end repair. Prior to MeDIP, the DNA was denatured in a 100°C heat block for 10 min and snap-cooled on ice for 5 min. The cooled single-stranded DNA was immunoprecipitated overnight on a rotator at 4°C with 1 μl of anti-5me-cytidine antibody (Diagenode, #MAb-5MECYT-100) in immunoprecipitation buffer (100 mM Na-Phosphate, pH 7.0; 1.4 M NaCl; 0.5% Triton X-100). Bound DNA was precipitated with sheep anti-mouse IgG Dynabeads (M-280, Invitrogen). The bound DNA was washed thrice with immunoprecipitation buffer for 10 min at room temperature with shaking, resuspended in 250 μl proteinase K digestion buffer (5 mM Tris, pH 8.0; 1 mM EDTA, pH 8.0; 0.05% SDS) with 7 μl of 10 mg/ml proteinase K and incubated for 3 hrs on an end-over-end rotator at 50°C to digest the antibodies and release the 5meC-containing DNA. The DNA was extracted once with 250 μl phenol, once with 250 μl chloroform and precipitated by adding 500 μl ethanol with 400 mM NaCl. To improve recovery, 1 μl glycogen (20 mg/ml) was added. DNA pellets were washed with 70% ethanol, resuspended in 50 μl TE buffer and stored at -20°C.

Immunoprecipitated DNA was tested for enrichment of methylated regions by duplex PCR targeting genomic regions expected to be differentially methylated. The expected methylated target was a putative retroelement (Poptr1_1/LG_XV:6357939-6358210, Additional file 2). The expected unmethylated target was a histone H2B gene (Poptr1_1/LG_II:21650848-21651585, Additional file 3). Relative enrichment was assessed qualitatively by brightness of bands on an electrophoretic gel.

### Illumina sequencing library preparation

The immunoprecipitated DNA was amplified by PCR with primers PE_PCR1.0 PE_PCR2.0 (Additional data file 3) to produce sequencing libraries. The number of PCR cycles required to produce a library for Illumina sequencing of recovered DNA was determined by testing a range of cycle numbers (15, 18, 21 cycles). For each library, three separate 20 μl PCRs with the appropriate number of cycles were combined. DNA was purified on a Qiagen PCR purification column (final elution volume of 52 μl TE buffer). DNA samples were quantified using a nanodrop ND-1000 spectrophotometer, then diluted to 10 nM for sequencing on an Illumina 1G or GAIIx Genome Analyzer.

### Bisulfite Sequencing

One microgram of genomic DNA from three biological replicates of each of three tissue types (autumn buds, winter buds, spring buds) was bisulfite-treated following the instructions included with the EpiTect Bisulfite kit (Qiagen). Prior to bisulfite treatment, aliquots from genomic DNA samples representing three biological replicates of each bud stage were pooled in equimolar amounts to serve as an untreated control. Targets for bisulfite sequencing were chosen to represent a variety of MeDIP-seq coverage levels (Additional file 2). The bisulfite-sequencing targets chosen for this study had a cytosine content ranging from 7.1%-24.0%. PCR primers were designed with Primer3 software, manually selecting regions with few cytosine bases in order to minimize primer degeneracy. PCRs were performed with Platinum *Taq *DNA polymerase (Invitrogen) in 25 μl reaction volumes containing 100 ng template DNA and 10 ng of each primer. PCR products were cloned following instructions included in TOPO TA cloning kits (Invitrogen). Ten clones amplified and isolated for each target region were sequenced from each of the three tissue types, and four clones were sequenced from the untreated pool. Sequences were aligned using ClustalW. Cytosine context was tallied and averaged for each set of clones.

### Bioinformatic Processing and Statistical Analyses

Illumina 40- or 36-nt sequencing reads were trimmed to a length 32 nt. Where reads were identical ("clonal reads"), all but one was removed (Additional files 15, 16). Reads were then aligned to the *P. trichocarpa *V2.2 reference genome and the *P.trichocarpa *chloroplast genome http://genome.ornl.gov/poplar_chloroplast/ with Eland http://www.illumina.com/Documents/products/datasheets/datasheet_genomic_sequence.pdf and HashMatch [67]. Eland alignments were performed using default parameters, which allow two mismatches per 32mer read. HashMatch alignments require perfect matches. Reads that aligned to the chloroplast or mitochondrial genomes (allowing up to two mismatches) were removed unless they were also perfect matches to the nuclear genome. Eland alignments were used to calculate the overall coverage per nucleotide as a measure for depth of sequencing for reads that align at unique positions, again allowing up to two mismatches. In a separate but parallel process, HashMatch was used to identify reads that align to multiple locations. These k-mer repeat reads were randomly and equally divided among all locations to which they aligned (allowing decimals) and coverage per nucleotide was calculated. Uniquely aligning reads were excluded from this branch of the pipeline which we refer to as "distributed" and/or "k-mer" repeats (Additional file 15). Sequencing depth or "coverage" was quantified by calculating Reads Per Kilobase of target sequence per Million reads mapped (RPKM; [68]). The RPKM measure was applied to one kilobase (kb) windows tiled across the entire genome, which was comprised of 378,538 windows. To check for potential bias toward cytosine-rich regions, numbers of cytosines per window were tallied, and these were compared to RPKM calculations (Additional data file 17); however, no relationship of RPKM to cytosine density was observed.

To study methylation-gene expression associations, RPKM was determined for specific features associated with annotated gene models (promoter, 5'UTR, gene body, exon, introns, 3'UTR, intergenic regions). To determine windows with RPKM that was statistically above that of the non-immunoprecipitated control (input), a false discovery rate (FDR) was calculated from 1-kb tiled windows for four lanes of input and the values for all MeDIP tissues pooled (sample). The arithmetic difference between input lanes was calculated and the distribution of these differences was determined for all possible permutations of input-input differences, and the mean of these distributions calculated. This process was repeated for the differences obtained from subtracting the average of all four input lanes from the sample. These distributions of differences were used to determine an RPKM cutoff that resulted in 100 significant windows in the sample-input comparison for every one significant window in the input-input comparison, thus a 1% FDR. By this procedure we arrived at an RPKM cutoff of 4.83. Genome feature context (promoter, intron, intergenic, etc.) was assigned to the collection of methylated windows. The results of this analysis were used for all tests of the relationship of methylation to expression, and descriptions of tissue-specific methylation patterns.

As alternative methods to quantify enrichment, we calculated the number of reads aligning in 1-kb windows with significant enrichment in MeDIP counts compared to input based on Poisson and negative binomial distributions. First, we normalized input counts to those of each MeDIP sample. For each window, if the input counts fell below the average input count for all windows, the counts were reset to their average value. We next used the cumulative Poisson probability distribution (CPPD) to estimate the probability of observing equal or greater read counts in the MeDIP sample than in the same window in the normalized input sample. Windows with probability of counts less than 0.0001 were considered significant (comparisons of input samples showed that this method yielded approximately a 1% FDR).

Finally, we used a negative binomial distribution to estimate the statistical significance of the peaks. The motivation for the use of the negative binomial is that it is a two parameter distribution that, unlike the Poisson where the mean and variance are equal, allows us to fit the observed variance of the data. We had observed that the variance across biological replicates was often larger than the mean, and therefore was not consistently fit by a Poisson distribution. This same observation has been made for RNA-seq data, where the use of negative binomials to estimate the significance of differential counts between a gene in different samples has become standard [69]. The implementation of this approach was in all other ways identical to the Poisson method described above, except that the probability of observing the MeDIP counts in a window, compared to those of the input samples, was estimated using the negative binomial distribution. The parameters of the negative binomial distribution for each tissue were fit by measuring the variance in our data across biological replicates using the Matlab function nbinfit. The p-value for each window was then estimated using the Matlab function nbincdf which computes the distribution of the cumulative negative binomial distribution. The first pass of this analysis used a p-value cutoff of 10^-4^, which corresponded to an estimated false discovery rate of 5% based on variation among biological replicates of the bud tissue samples. These parameters called only 653 windows methylated in all tissues, 326,478 windows non-methylated in all tissues, and 51,405 windows differentially methylated among tissues. In a second pass, a peak was called in each window that had a p-value of 10^-3^, which corresponded to an estimated false discovery rate of 20%, also based on biological replicates of the bud tissue samples. Using the results of this analysis, the agreement among the three methods was calculated by dividing the number of methylated windows called by all of the methods by the total number of windows called by any of the methods. Genes with methylation at promoters, and/or within annotated transcribed regions (gene bodies) were compared to archival expression microarray data to determine correlation between methylation and expression. Mann-Whitney tests were used to compare RPKM or gene expression of groups of genes among tissues assuming independence of genes within biological replicates, and Sign tests [70] were used to evaluate the statistical significance of consistency among tissues in genic methylation and expression patterns.

Enrichment of gene ontology (GO) categories within sets of methylated genes was tested using the AgriGO singular enrichment analysis tool applied to the Poplar v2.2 genome reference gene ontology set, using default parameters except for the selection of the Bonferroni multiple-test correction method: http://bioinfo.cau.edu.cn/agriGO/analysis.php. GO enrichments were visualized using Cytoscape v.1.4. http://www.cytoscape.org. The RPKM data are available for browsing and downloading using Gbrowse version 2.13 at http://http:poplar.cgrb.oregonstate.edu. http://gmod.org. All MeDIP-seq data were submitted to the National Center for Biotechnology Information (NCBI) Sequence Read Archive (SRA) database (accession #SRA039208.1).

## Competing interests

The authors declare that they have no competing interests.

## Authors' contributions

Conceived and designed the experiments: KV KP SS TM MF. Performed the experiments: KV KP Analyzed the data: KV LW KP HP MP TM. Contributed reagents/materials/analysis tools: LW HP TM MF MP. Wrote the paper: KV KP MF LW TM SS. All authors read and approved the final manuscript.

## Supplementary Material

Additional file 1 **Images of tissues sampled in this study**. Click here for file

Additional file 2 **Bisulfite sequencing targets, annotations**. Click here for file

Additional file 3 **Primers used in this study**. Click here for file

Additional file 4 **Percentage methylated cytosines in bisulfite-sequencing targets in relationship to RPKM and maximum per-nucleotide MeDIP-seq coverage from genomic data**. Average percentage methylated cytosines was calculated for eight targets PCR-amplified from three bisulfite-treated bud types. A. Percentage methylated cytosines plotted against RPKM calculated for each target region. B. Percentage methylated cytosines plotted against maximum per-nucleotide coverage within target region. Click here for file

Additional file 5 **Methylated cytosine context in bisulfite-sequencing targets**. Percentage of methylated cytosines is shown for eight target regions amplified from bisulfite-treated DNA from each of three bud stages. Cytosines as a percentage of the total number bases in each target is shown for the control (not bisulfite-treated) sample. Cytosines in each of the three sequence contexts are shown as percentages of the total number of cytosines. Click here for file

Additional file 6 **Example of cytosine methylation differentiation in a bisulfite sequencing target**. Ten cloned PCR products were aligned for each of three bud stages (fall, winter, spring). Each square represents a cytosine base in the sequence of a unique cloned sequence. Not the variation in consistency among methylation context types. Click here for file

Additional file 7 **Depth of MeDIP-seq genome coverage**. Bars represent the portion of the *P. trichocarpa *V2.2 genome covered by MeDIP sequence data, organized by tissue type. "Input" is constituted of three sequencing lanes of a non-immunoprecipitated control sample. Darker bars indicate genome coverage by uniquely-mapping reads, while lighter upper parts of bars show genome coverage by reads that mapped to more than one position and were equally distributed over all genome occurrences. Uniquely-mapping reads and distributed k-mer repeats are not mutually exclusive, in that reads of the two different types may partially overlap. A. Overall genome coverage by MeDIP-seq reads. B. Average per-nucleotide sequence depth. Click here for file

Additional file 8 **Chromosome view of methylation in relation to gene and k-mer repeat density**. MeDIP-seq reads were aligned to each of the 19 *P. trichocarpa *chromosomes. Asterisks mark putative centromeres for chromosomes where a single centromeric locus seems to be clear. Blue (above lines) = gene density. Black (below lines) = unique Me-DIP reads. Red (below line) = k-mer distributed MeDIP repeats. Click here for file

Additional file 9 **Regions of chromosomes with strong differences in methylation among tissues**. Counts of MeDIP-seq reads were plotted in 1 kb windows along chromosomes. One line is shown for each tissue type. A. Zooming in on a region of chromosome 10 (dashed line) shows decreased methylation in female catkins relative to other tissues over a gene-poor, transposable-element-rich region. B. Zooming in on a region of chromosome 19 (dashed line) shows decreased methylation in male and female catkins relative to other tissues over a gene-poor, transposable-element-rich region. Click here for file

Additional file 10 **MeDIP sequence coverage of genomic features**. Bars show the percent of each feature type with a non-zero RPKM value. RPKM values were calculated based on the feature width as defined in version 2.2 of the *P. trichocarpa *genome annotation. Promoters were defined as the 2 kb region upstream of the annotated transcription start site. Intergenic spaces were divided into 1 kb windows. The RepPop repetitive element feature includes all entries in the RepPop database. A) Uniquely-aligning reads; B) Distributed k-mer repeats. Click here for file

Additional file 11 **Association of methylation level with variation in methylation among tissues**. Average RPKM across all tissue types was calculated for 378,536 1 kb windows covering the *P. trichocarpa *genome and plotted against its standard deviation based on tissue means. Using General Linear Model regression analysis, R^2 ^= 0.68, P < 2e-16. Click here for file

Additional file 12 **ver-represented gene ontology categories in genes with significantly methylated gene bodies in male catkins and no other tissues**. Circles are shaded based on significance level (yellow = FDR < 0.05), and the radius of each circle is proportional to the number of genes in each category. Click here for file

Additional file 13 **Clustering of tissues based on gene expression**. Matrix-based hierarchical clustering was performed using the iterative R hclust function with the default complete linkage method. The diagram shows correlation of Nimblegen array expression data among tissue types. Click here for file

Additional file 14 **Clustering of tissues based on RPKM values for 1 kb genome windows**. Hierarchical clustering of biological replicates from all samples. Distance matrices were based on Pearson correlation of RPKM counts of methylated 1 kb windows. Click here for file

Additional file 15 **Bioinformatic processing pipeline**. The pipeline shows processing steps from initial read-filtering and normalization through identification of methylated genome features to downstream display and download capabilities. Click here for file

Additional file 16 **Non-clonal read frequency in relation to the number of library amplification cycles**. Each bar represents one MeDIP-seq lane. Letters after tissue labels designate biological replicates within tissue types. A. Clonal read frequency. B. Illumina sequencing library PCR amplification cycles. Asterisks indicate libraries for which 18-cycle and 21-cycle amplification products were mixed. Click here for file

Additional file 17 **Relationship of cytosine content to RPKM**. The number of cytosines was tallied for each 1 kb genome window, and the collection of windows was then divided into deciles. Each box represents one decile, lowest to highest cytosine content moving left to right. There was no apparent relationship between cytosine density and RPKM. Click here for file
