# Supplementary figures and images for: Dynamic DNA cytosine methylation in the Populus trichocarpa genome: tissue-level variation and relationship to gene expression
Source: BMC Genomics. 2012 Jan 17;13:27. doi: 10.1186/1471-2164-13-27 (PMC3298464; doi:10.1186/1471-2164-13-27)

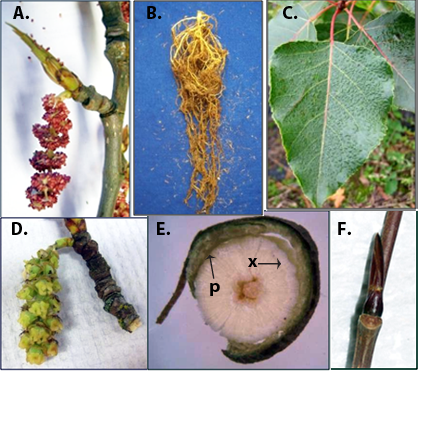

Supplement: Additional file 1 — Images of tissues sampled in this study. [file 1471-2164-13-27-S1.TIFF]

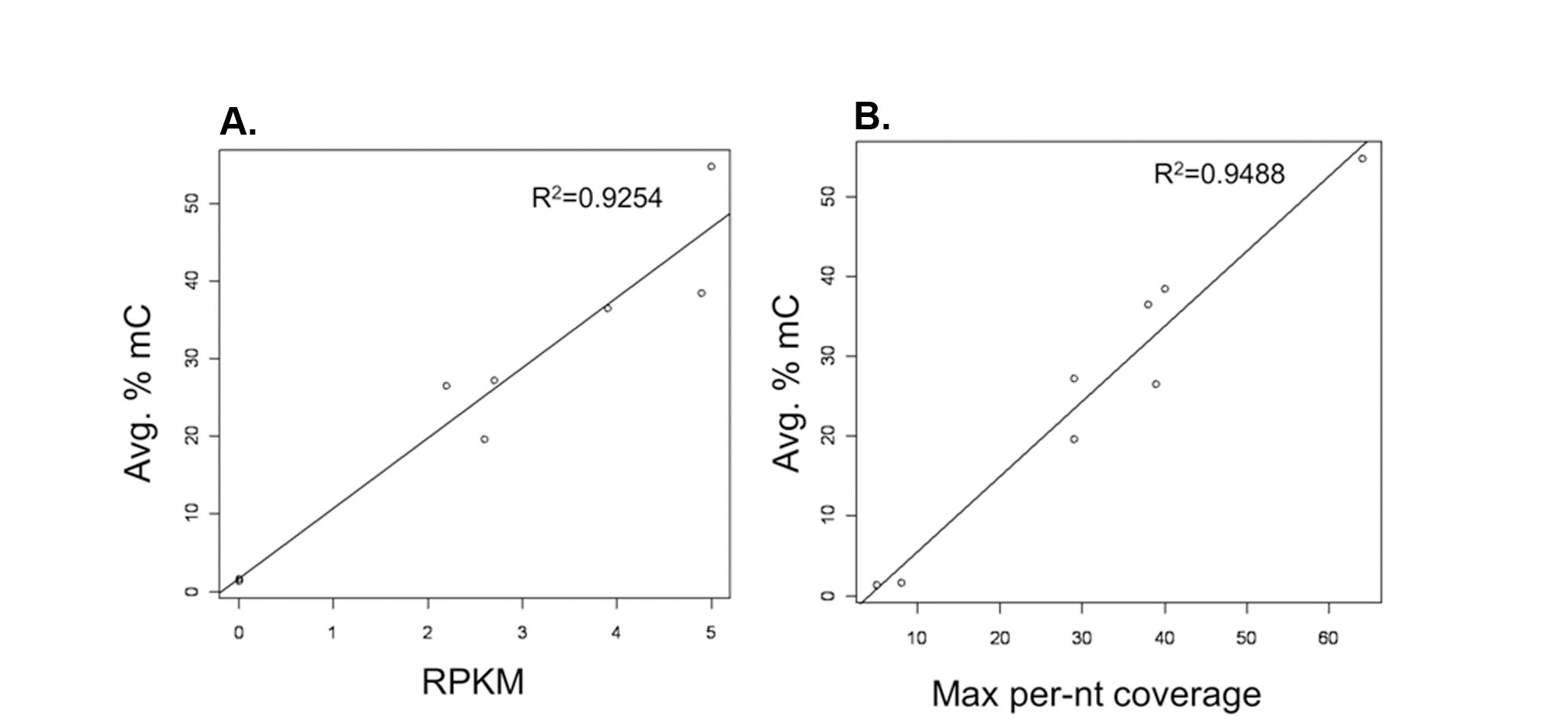

Supplement: Additional file 4 — Percentage methylated cytosines in bisulfite-sequencing targets in relationship to RPKM and maximum per-nucleotide MeDIP-seq coverage from genomic data. Average percentage methylated cytosines was calculated for eight targets PCR-amplified from three bisulfite-treated bud types. A. Percentage methylated cytosines plotted against RPKM calculated for each target region. B. Percentage methylated cytosines plotted against maximum per-nucleotide coverage within target region. [file 1471-2164-13-27-S4.TIFF]

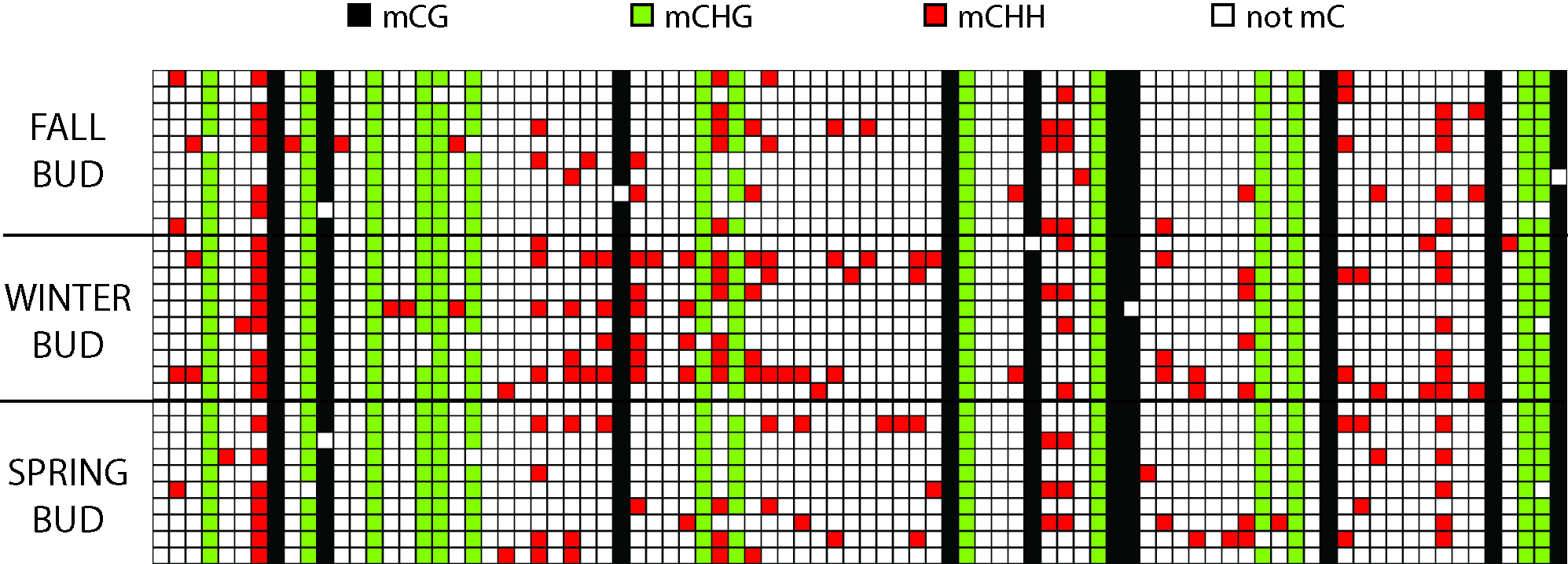

Supplement: Additional file 6 — Example of cytosine methylation differentiation in a bisulfite sequencing target. Ten cloned PCR products were aligned for each of three bud stages (fall, winter, spring). Each square represents a cytosine base in the sequence of a unique cloned sequence. Not the variation in consistency among methylation context types. [file 1471-2164-13-27-S6.TIFF]

**A.**

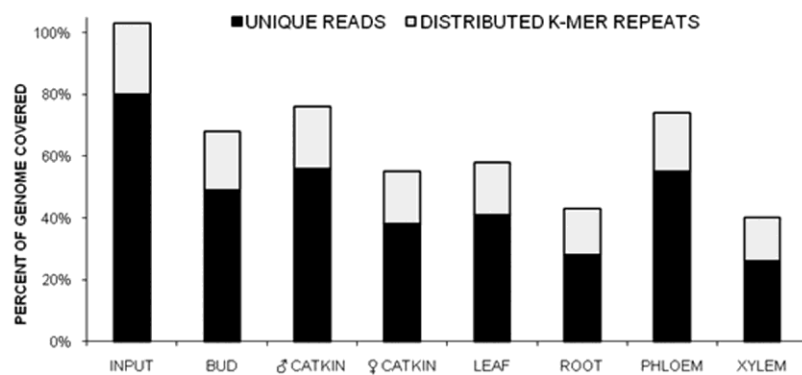

**B.**

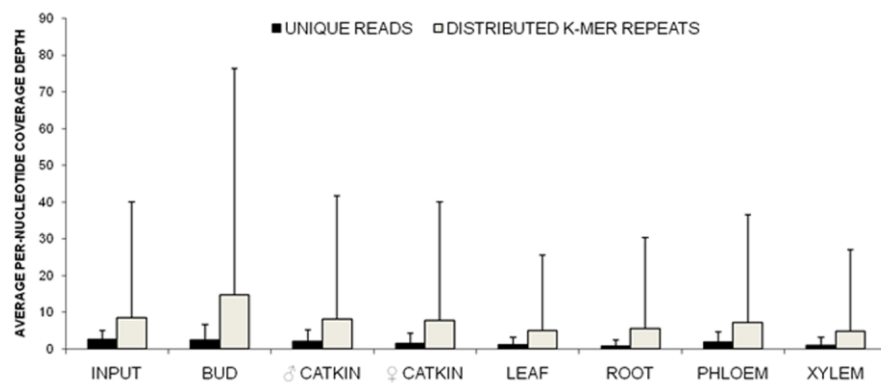

Supplement: Additional file 7 — Depth of MeDIP-seq genome coverage. Bars represent the portion of the P. trichocarpa V2.2 genome covered by MeDIP sequence data, organized by tissue type. "Input" is constituted of three sequencing lanes of a non-immunoprecipitated control sample. Darker bars indicate genome coverage by uniquely-mapping reads, while lighter upper parts of bars show genome coverage by reads that mapped to more than one position and were equally distributed over all genome occurrences. Uniquely-mapping reads and distributed k-mer repeats are not mutually exclusive, in that reads of the two different types may partially overlap. A. Overall genome coverage by MeDIP-seq reads. B. Average per-nucleotide sequence depth. [file 1471-2164-13-27-S7.PDF]

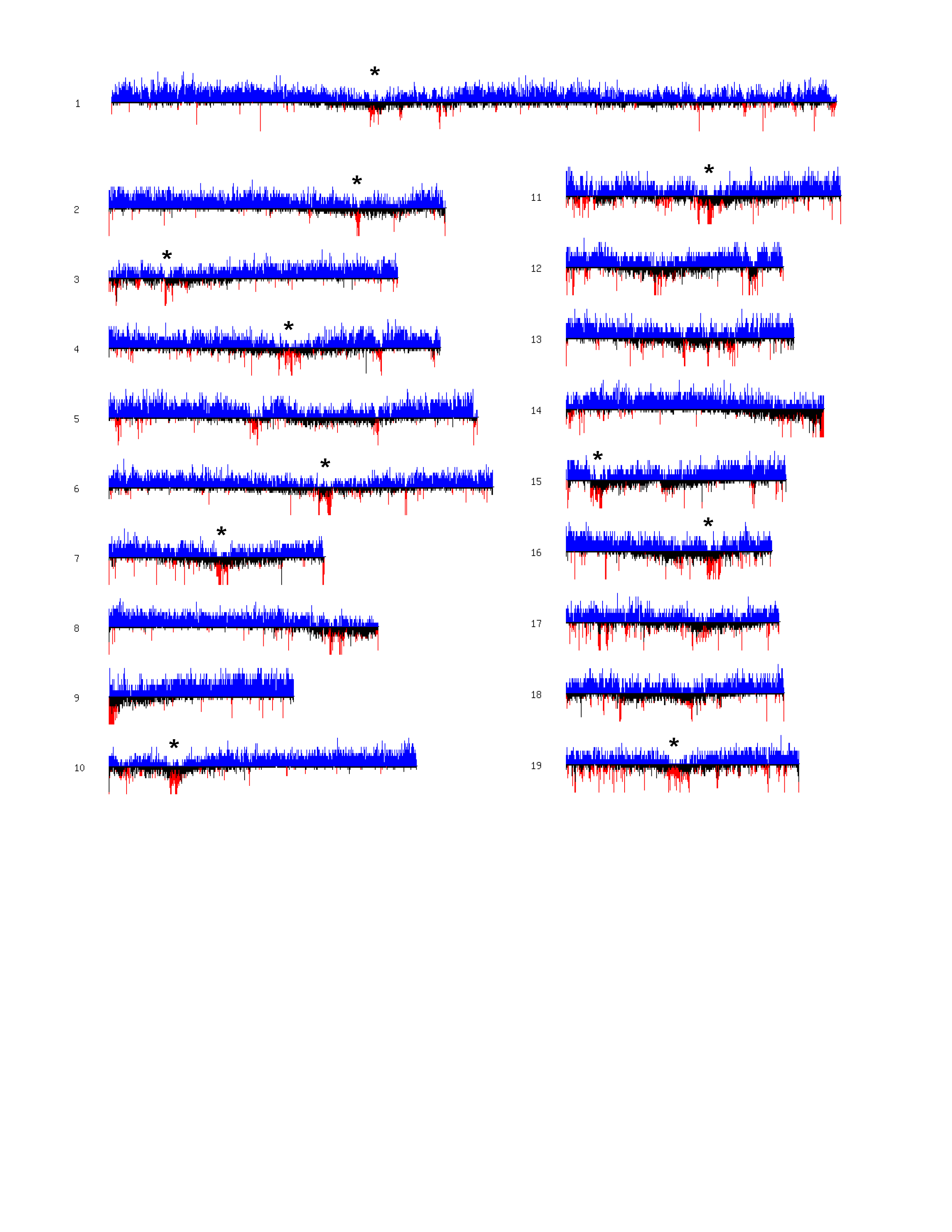

Supplement: Additional file 8 — Chromosome view of methylation in relation to gene and k-mer repeat density. MeDIP-seq reads were aligned to each of the 19 P. trichocarpa chromosomes. Asterisks mark putative centromeres for chromosomes where a single centromeric locus seems to be clear. Blue (above lines) = gene density. Black (below lines) = unique Me-DIP reads. Red (below line) = k-mer distributed MeDIP repeats. [file 1471-2164-13-27-S8.TIFF]

A.

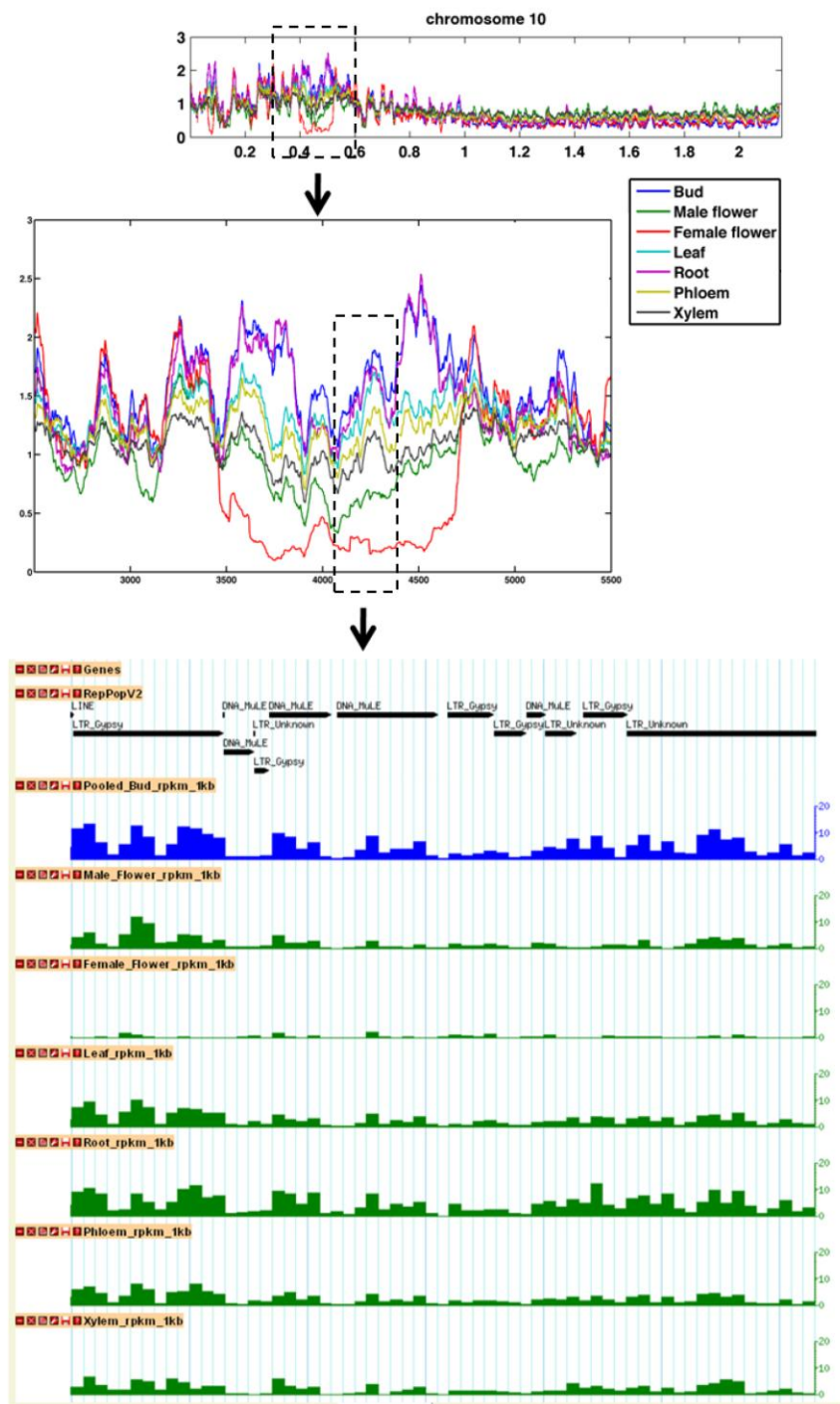

B.

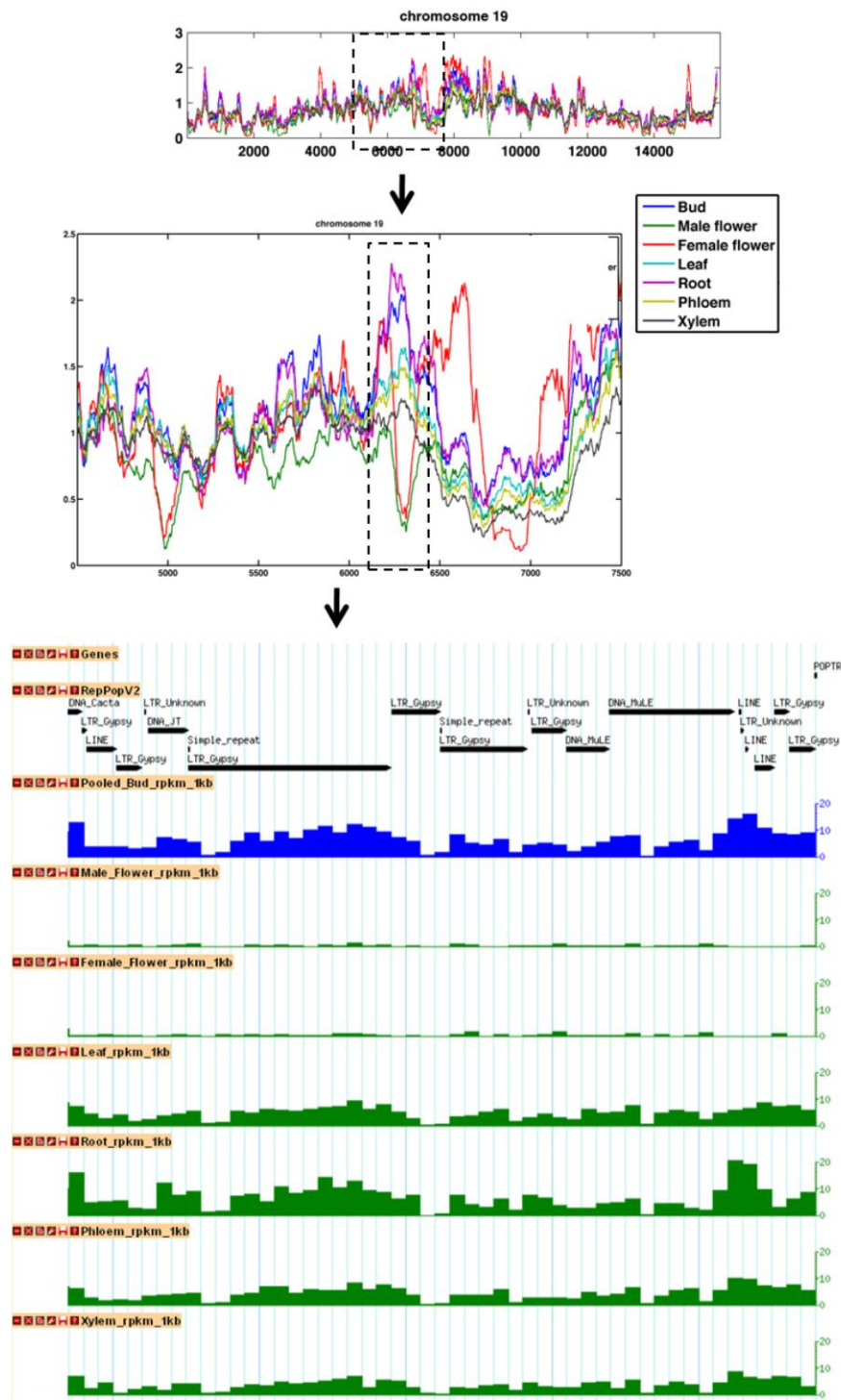

Supplement: Additional file 9 — Regions of chromosomes with strong differences in methylation among tissues. Counts of MeDIP-seq reads were plotted in 1 kb windows along chromosomes. One line is shown for each tissue type. A. Zooming in on a region of chromosome 10 (dashed line) shows decreased methylation in female catkins relative to other tissues over a gene-poor, transposable-element-rich region. B. Zooming in on a region of chromosome 19 (dashed line) shows decreased methylation in male and female catkins relative to other tissues over a gene-poor, transposable-element-rich region. [file 1471-2164-13-27-S9.PDF]

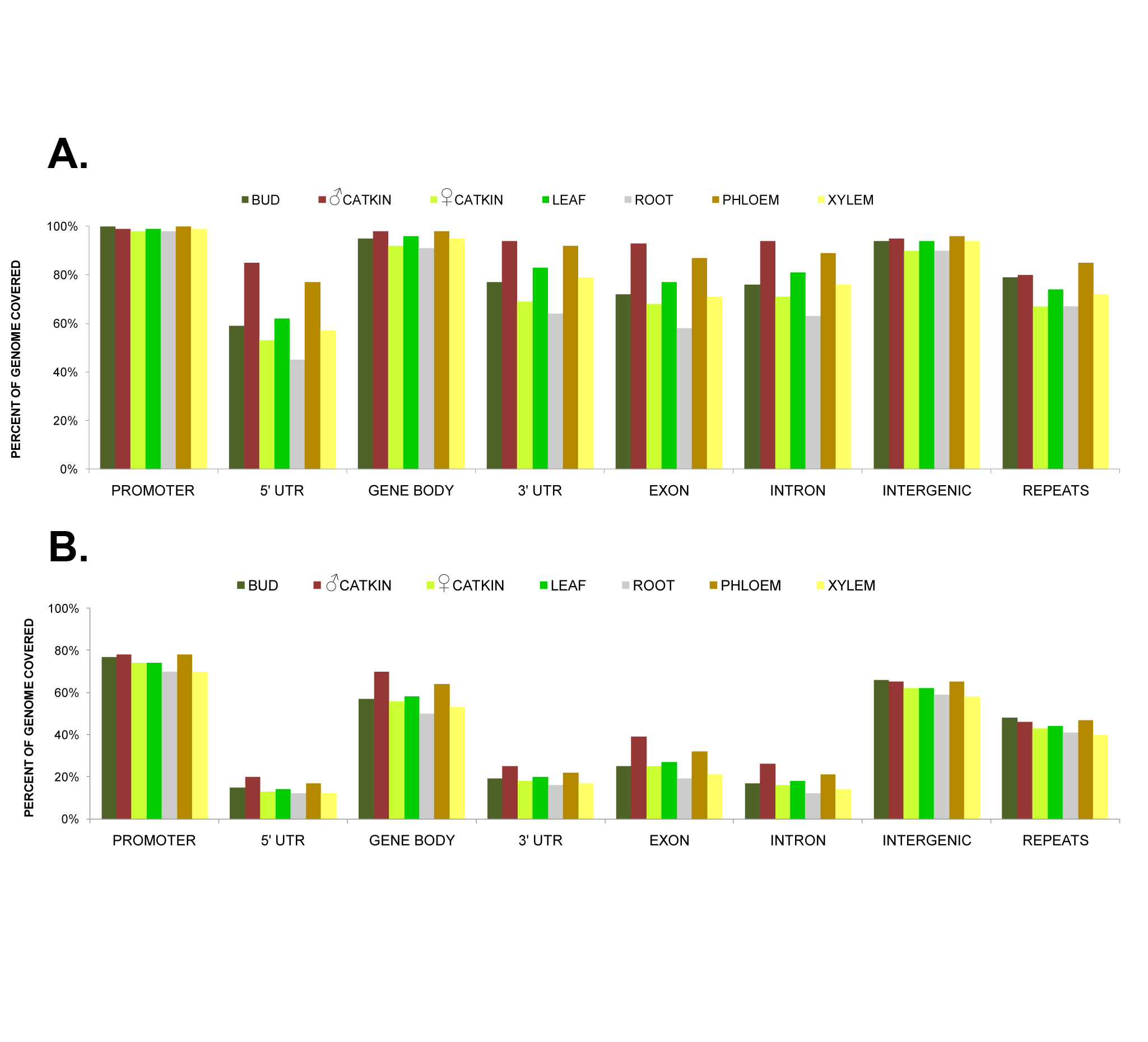

Supplement: Additional file 10 — MeDIP sequence coverage of genomic features. Bars show the percent of each feature type with a non-zero RPKM value. RPKM values were calculated based on the feature width as defined in version 2.2 of the P. trichocarpa genome annotation. Promoters were defined as the 2 kb region upstream of the annotated transcription start site. Intergenic spaces were divided into 1 kb windows. The RepPop repetitive element feature includes all entries in the RepPop database. A) Uniquely-aligning reads; B) Distributed k-mer repeats. [file 1471-2164-13-27-S10.TIFF]

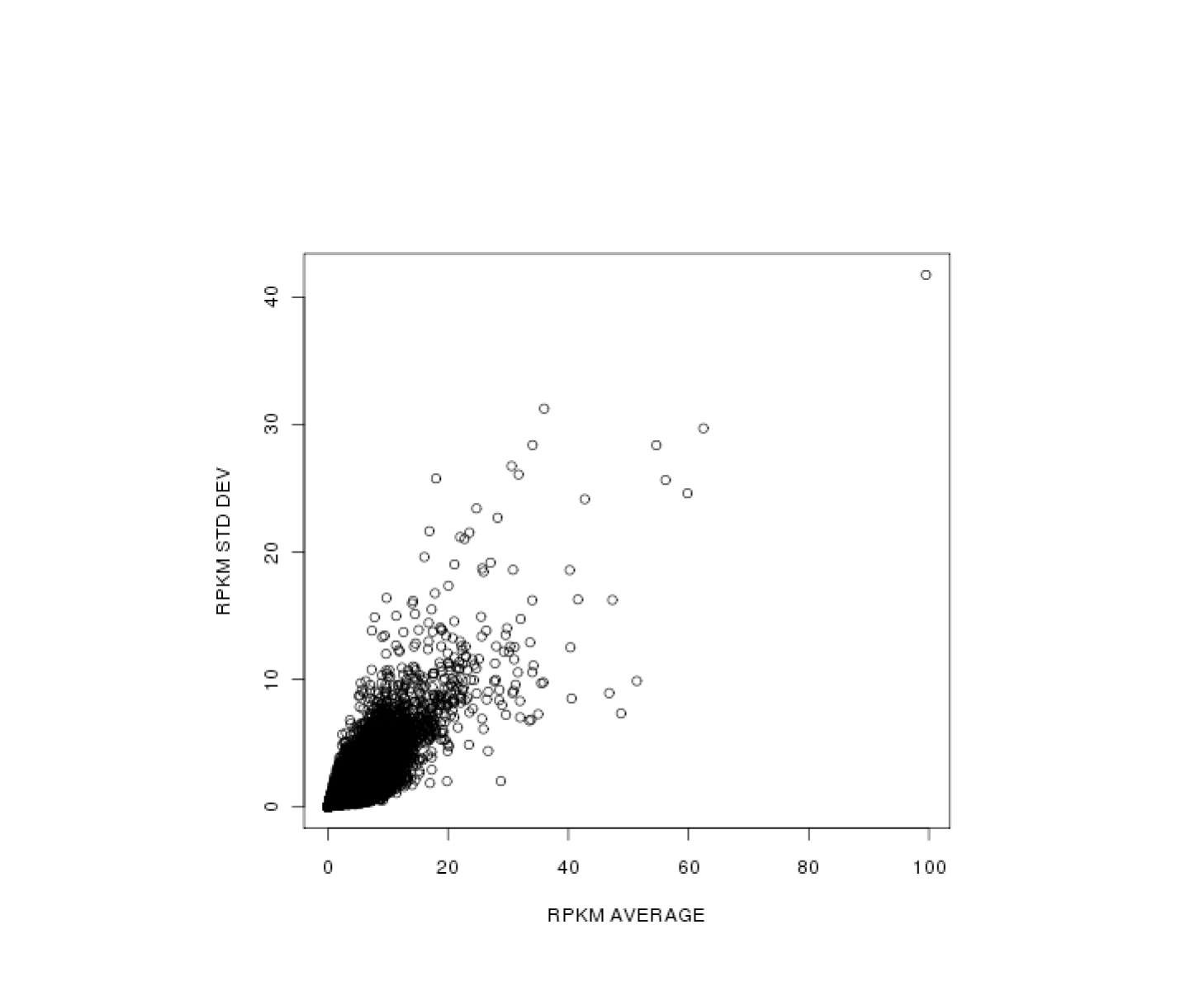

Supplement: Additional file 11 — Association of methylation level with variation in methylation among tissues. Average RPKM across all tissue types was calculated for 378,536 1 kb windows covering the P. trichocarpa genome and plotted against its standard deviation based on tissue means. Using General Linear Model regression analysis, R2 = 0.68, P < 2e-16. [file 1471-2164-13-27-S11.TIFF]

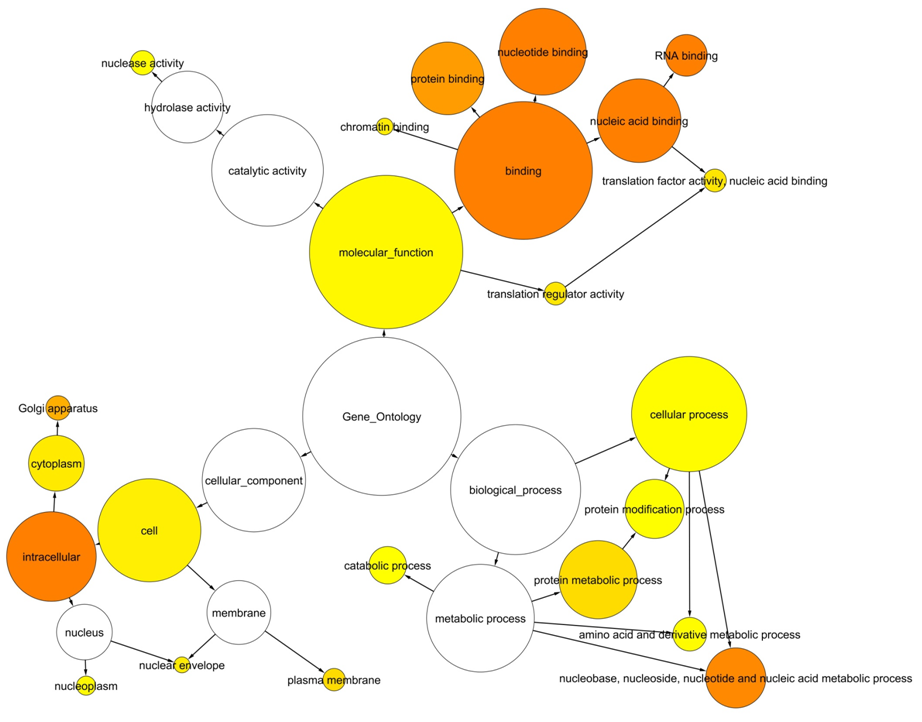

Supplement: Additional file 12 — ver-represented gene ontology categories in genes with significantly methylated gene bodies in male catkins and no other tissues. Circles are shaded based on significance level (yellow = FDR < 0.05), and the radius of each circle is proportional to the number of genes in each category. [file 1471-2164-13-27-S12.TIFF]

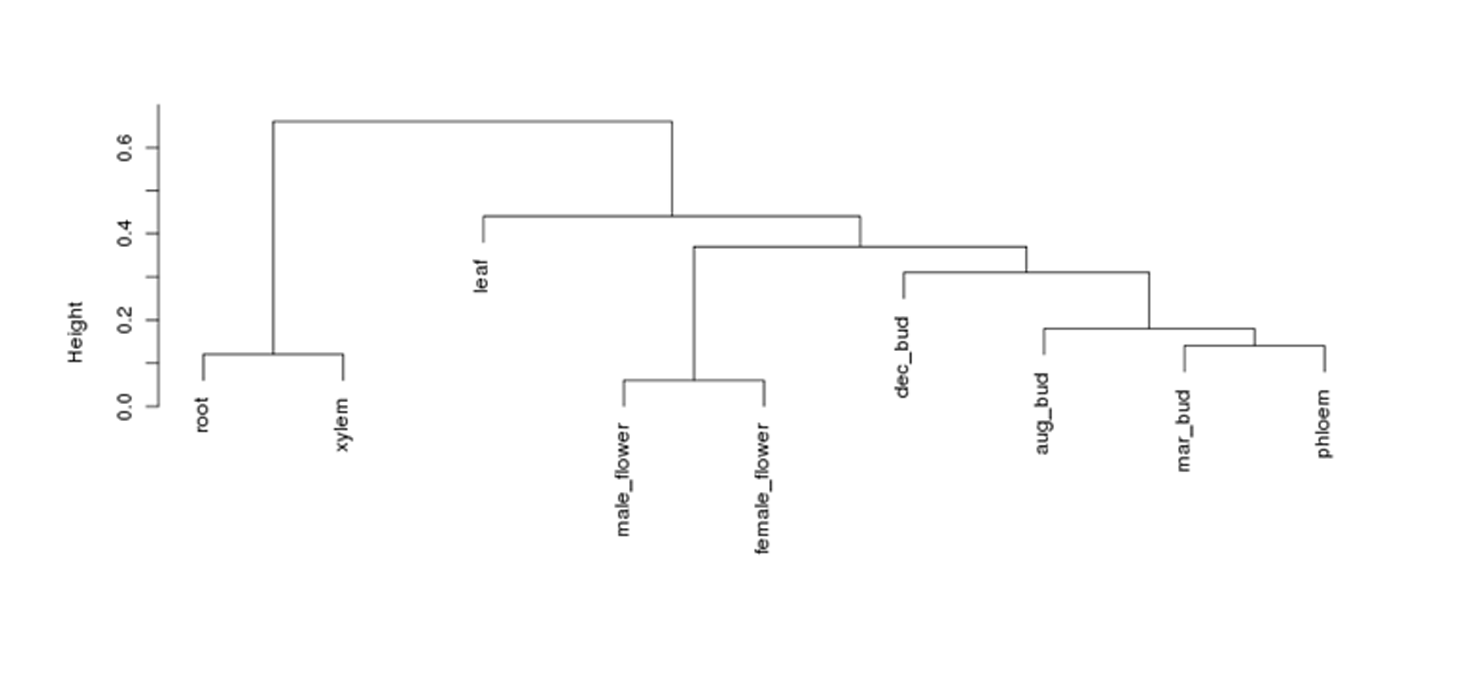

Supplement: Additional file 13 — Clustering of tissues based on gene expression. Matrix-based hierarchical clustering was performed using the iterative R hclust function with the default complete linkage method. The diagram shows correlation of Nimblegen array expression data among tissue types. [file 1471-2164-13-27-S13.TIFF]

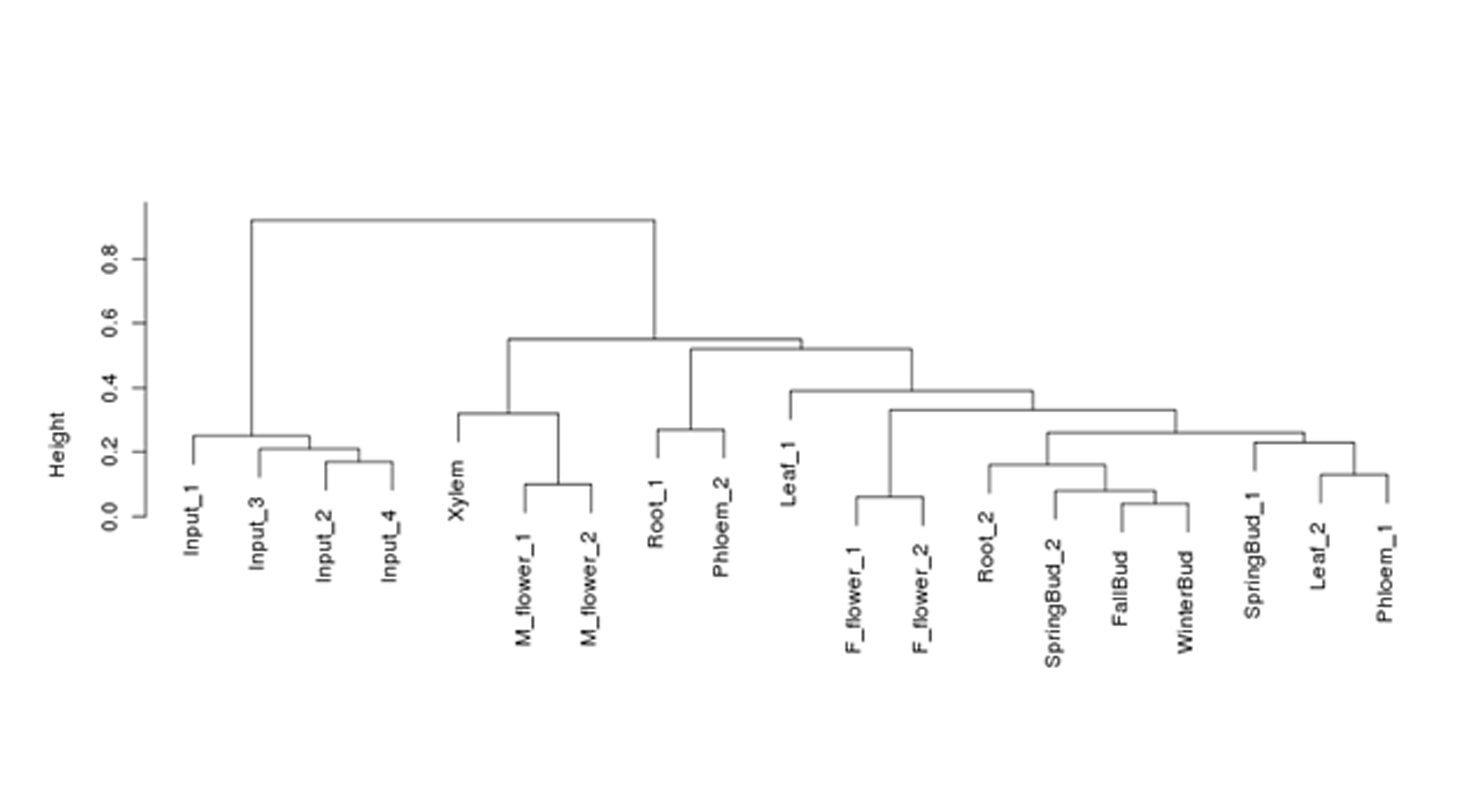

Supplement: Additional file 14 — Clustering of tissues based on RPKM values for 1 kb genome windows. Hierarchical clustering of biological replicates from all samples. Distance matrices were based on Pearson correlation of RPKM counts of methylated 1 kb windows. [file 1471-2164-13-27-S14.TIFF]

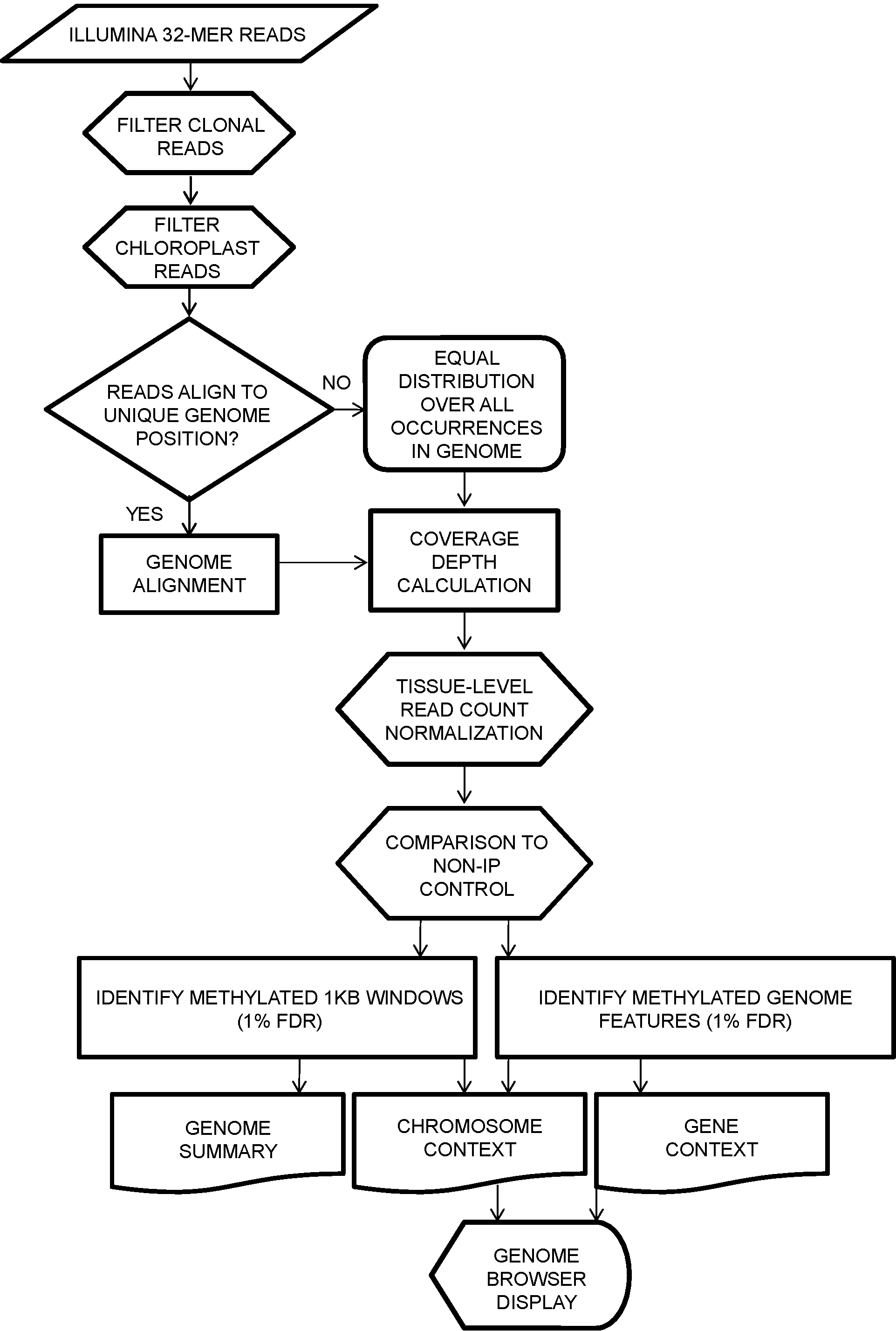

Supplement: Additional file 15 — Bioinformatic processing pipeline. The pipeline shows processing steps from initial read-filtering and normalization through identification of methylated genome features to downstream display and download capabilities. [file 1471-2164-13-27-S15.TIFF]

A.

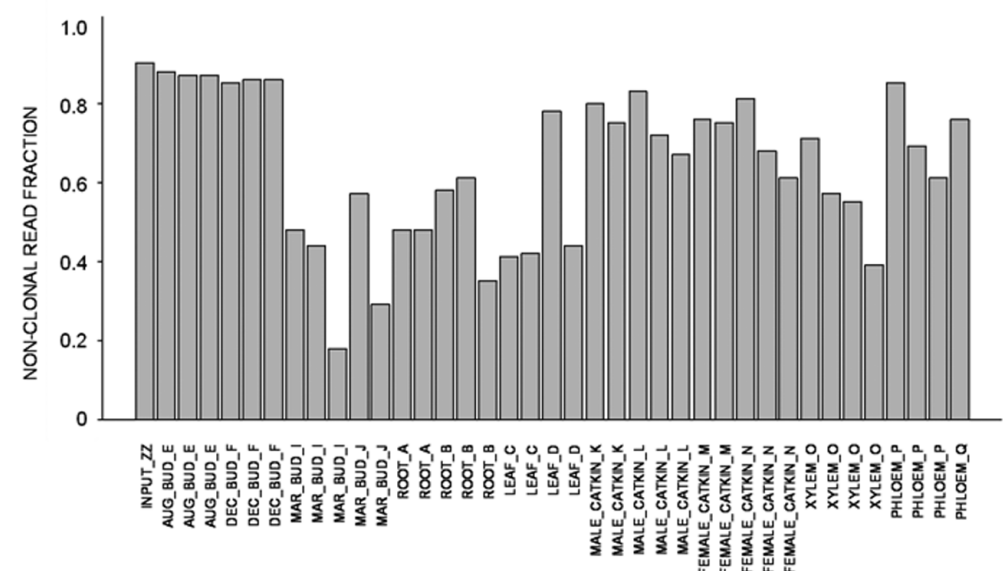

B.

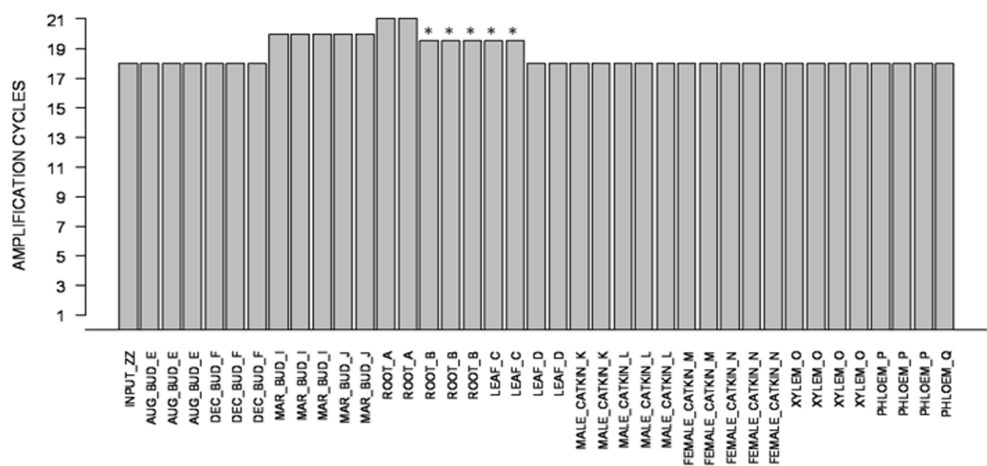

Supplement: Additional file 16 — Non-clonal read frequency in relation to the number of library amplification cycles. Each bar represents one MeDIP-seq lane. Letters after tissue labels designate biological replicates within tissue types. A. Clonal read frequency. B. Illumina sequencing library PCR amplification cycles. Asterisks indicate libraries for which 18-cycle and 21-cycle amplification products were mixed. [file 1471-2164-13-27-S16.PDF]
