# Supplementary material for: Dynamic DNA cytosine methylation in the Populus trichocarpa genome: tissue-level variation and relationship to gene expression
Source: BMC Genomics. 2012 Jan 17;13:27. doi: 10.1186/1471-2164-13-27 (PMC3298464; doi:10.1186/1471-2164-13-27)
Supplement: Additional file 2 — Bisulfite sequencing targets, annotations. [file 1471-2164-13-27-S2.DOC]

**Additional file 2: Bisulfite sequencing targets, annotations.**

| **Target local name** | **Target location** | **Gene_model** | **scaffold** | **start** | **end** | **size** | **RPKM** | **max_**  **per_nt MeDIP_**  **cov** | **avg_%mC from bisulf-seq** | **Annotation** |
| --- | --- | --- | --- | --- | --- | --- | --- | --- | --- | --- |
| **984F** | 5' of gene | POPTR_0010s18680.1 | 10 | 4667177 | 4667857 | 680 | 3.9 | 38 | 36.5% | glycine/proline-rich protein |
| **2035F** | 5'/coding | POPTR_0004s17990.1 | 4 | 18014021 | 18014540 | 519 | 5.0 | 64 | 54.7% | ubiquitin extension protein, putative / 40S ribosomal protein S27A (RPS27A) |
| **E09_E10** | 5' of gene | POPTR_0001s16990.1 | 1 | 13807422 | 13808119 | 697 | 4.9 | 40 | 38.5% | SNF2 domain-containing protein / helicase domain-containing |
| **G07_G06** | 5' of gene | POPTR_0002s13480.1 | 2 | 10010714 | 10011173 | 459 | 0.0 | 5 | 1.3% | BZR1 (BRASSINAZOLE-RESISTANT 1) |
| **F12_G01** | 5' of gene | POPTR_0003s04700.1 | 3 | 5685146 | 5685507 | 361 | 2.6 | 29 | 19.6% | ATHWHY2 (A. THALIANA WHIRLY 2) |
| **F01_F02** | 5'/coding | POPTR_0017s08090.1 | 17 | 6488866 | 6489228 | 362 | 0.0 | 8 | 1.7% | ethylene-responsive element-binding family protein |
| **F07_F08** | 5' of gene | POPTR_0014s006890.1 | 14 | 647831 | 648406 | 575 | 2.7 | 29 | 30.0% | WRKY21 (WRKY DNA-binding protein 21) |
| **G08_G09** | 5' of gene | POPTR_0003s1730.1 | 3 | 1598895 | 1599251 | 356 | 2.2 | 39 | 26.5% | XYL4 (beta-xylosidase 4) |
