# Supplementary material for: Dynamic DNA cytosine methylation in the Populus trichocarpa genome: tissue-level variation and relationship to gene expression
Source: BMC Genomics. 2012 Jan 17;13:27. doi: 10.1186/1471-2164-13-27 (PMC3298464; doi:10.1186/1471-2164-13-27)
Supplement: Additional file 3 — Primers used in this study. [file 1471-2164-13-27-S3.DOC]

**Additional file 3. Primers used in this study.**

| **Target** | **Purpose** | **Forward primer (5' -> 3')** | **Reverse primer (5' -> 3')** |
| --- | --- | --- | --- |
| Poptr1_1/LG_XV:6357939..6358210 | 5mC enrichment, methylated target control | CAAACGCTACTCTGATGGACTTT | GGGGAAGATCAAGAGGTAGAGAC |
| Poptr1_1/LG_II:21650848-21651585 | 5mC enrichment, unmethylated target control | TCTTGATTAGCTATGTGCCTTTCTT | GAGTTGGAACCATAATTGAACAGAG |
| PE_PCR1.0_PCR2.0_Illumina_adaptors | Illumina sequencing library amplification | AATGATACGGCGACCACCGAGATCTACACTCTTTCCCTACACGACGCTCTTCCGATCT | CAAGCAGAAGACGGCATACGAGATCGGTCTCGGCATTCCTGCTGAACCGCTCTTCCGATCT |
| POPTR_0010s18680.1:4667177..4667857 | bisulfite sequencing | AAYGAGYTTGGAGGGTGAA | TRTTRRARCCAACCTARCCA |
| POPTR_0007s06970.1:5332787..5333347 | bisulfite sequencing | GGAGYATAYTGGGYAAAGTG | TCCTCCATCRTCAATRACTC |
| POPTR_0004s17990.1:18014021..18014540 | bisulfite sequencing | AYAAGGYAGAGATGGTGAT | CTRACTCCTCCTRAAT |
| POPTR_0001S16990.1:13807422..13808119 | bisulfite sequencing | AAACTAAACCCAACCRAACTCA | AGGAGTAYTGGGTTYGGAYATA |
| POPTR_0002s13480.1:10010714..10011173 | bisulfite sequencing | TATTTRTTRTTRTCRCCACCT | YAGGGAAGYAGAGAAGAGGA |
| POPTR_0003s04700.1:5685146..5685507 | bisulfite sequencing | TAACCCAARCTAAAACATCATA | GAGAGGAGTTAAYGYTGTTGT |
| POPTR_0017s08090.1:6488866..6489228 | bisulfite sequencing | CATCAAATTTRACARCCATATCTA | GAAAYAGGAGGGTAYTTGTGG |
| POPTR_0014s006890.1:647831..648406 | bisulfite sequencing | TYGAGGGAGAGGAATTGAGGA | ATAACCATCTATCCATCACRAA |
| POPTR_0003s1730.1:1598895..1599251 | bisulfite sequencing | RTTATRAAAACCCTCAAAACAA | YYGTGAAATTAGTYGAGGT |
