# Supplementary material for: Dynamic DNA cytosine methylation in the Populus trichocarpa genome: tissue-level variation and relationship to gene expression
Source: BMC Genomics. 2012 Jan 17;13:27. doi: 10.1186/1471-2164-13-27 (PMC3298464; doi:10.1186/1471-2164-13-27)
Supplement: Additional file 5 — Methylated cytosine context in bisulfite-sequencing targets. Percentage of methylated cytosines is shown for eight target regions amplified from bisulfite-treated DNA from each of three bud stages. Cytosines as a percentage of the total number bases in each target is shown for the control (not bisulfite-treated) sample. Cytosines in each of the three sequence contexts are shown as percentages of the total number of cytosines. [file 1471-2164-13-27-S5.DOC]

|  | |  |  |  |  |  |  |  |  |  |  |  |  |  |  |  |  |  |
| --- | --- | --- | --- | --- | --- | --- | --- | --- | --- | --- | --- | --- | --- | --- | --- | --- | --- | --- |
|  | **Target_**  **local_name** | | **2035F** |  |  |  | **E09_E10** | |  |  | **984F** |  |  |  | **F12_G01** |  |  |  |
|  | |  |  | **Length = 488bp** |  |  |  | **Length = 654 bp** | |  |  | **Length = 522 bp** | |  |  | **Length = 320 bp** | |  |
|  | |  | **MeDIP-seq RPKM = 5.0** | | | | **MeDIP-seq RPKM = 4.9** | | | | **MeDIP-seq RPKM = 3.9** | | | | **MeDIP-seq RPKM = 2.7** | | | |
|  | |  | **Total C** | **CG** | **CHG** | **CHH** | **Total C** | **CG** | **CHG** | **CHH** | **Total C** | **CG** | **CHG** | **CHH** | **Total C** | **CG** | **CHG** | **CHH** |
|  | | **Control** | **21.9%** | 7.5% | 16.8% | 75.7% | **24.0%** | 8.9% | 17.8% | 73.2% | **16.1%** | 13.1% | 16.7% | 71.4% | **16.1%** | 6.5% | 7.6% | 85.9% |
| **Bisulfite-treated** | | Fall_bud | **12.7%** | 14.7% | 32.3% | 53.0% | **7.4%** | 7.4% | 53.1% | 18.3% | **5.5%** | 36.2% | 47.8% | 15.9% | **5.9%** | 27.1% | 32.7% | 40.1% |
|  |  | Winter_bud | **13.0%** | 15.0% | 33.8% | 51.1% | **9.6%** | 9.6% | 43.0% | 35.2% | **6.3%** | 31.5% | 42.3% | 26.2% | **6.2%** | 21.5% | 34.2% | 44.4% |
|  |  | Spring_bud | **10.2%** | 19.8% | 43.8% | 36.3% | **10.7%** | 10.7% | 38.8% | 41.1% | **5.9%** | 16.5% | 45.5% | 21.4% | **4.8%** | 32.4% | 38.7% | 29.0% |
|  | |  |  |  |  |  |  |  |  |  |  |  |  |  |  |  |  |  |
|  |  |  |  |  |  |  |  |  |  |  |  |  |  |  |  |  |  |  |
|  |  |  |  |  |  |  |  |  |  |  |  |  |  |  |  |  |  |  |
|  | |  |  |  |  |  |  |  |  |  |  |  |  |  |  |  |  |  |
|  | |  |  |  |  |  |  |  |  |  |  |  |  |  |  |  |  |  |
|  | | **Target_local_name** | **F07_F08** |  |  |  | **G08_G09** | |  |  | **G07_G06** | |  |  | **F01_F02** |  |  |  |
|  | |  |  | **Length = 533 bp** | |  |  | **Length = 318 bp** | |  |  | **Length = 318 bp** | |  |  | **Length = 325 bp** | |  |
|  | |  | **MeDIP-seq RPKM = 2.6** | | | | **MeDIP-seq RPKM = 2.2** | | | | **MeDIP-seq RPKM = 0.0** | | | | **MeDIP-seq RPKM = 0.0** | | | |
|  | |  | **Total C** | **CG** | **CHG** | **CHH** | **Total C** | **CG** | **CHG** | **CHH** | **Total C** | **CG** | **CHG** | **CHH** | **Total C** | **CG** | **CHG** | **CHH** |
|  | | **Control** | **14.8%** | 21.5% | 27.8% | 50.6% | **13.5%** | 9.3% | 9.3% | 81.4% | **7.1%** | 12.9% | 25.8% | 61.3% | **8.9%** | 24.1% | 20.7% | 55.2% |
| **Bisulfite-treated** | | Fall_bud | **5.0%** | 62.2% | 25.0% | 12.9% | **3.9%** | 36.8 | 28.6% | 34.7% | **0.1%** | 0.0% | 0.0% | 100.0% | **0.2%** | 28.6% | 14.3% | 57.1% |
|  |  | Winter_bud | **4.1%** | 70.3% | 27.7% | 2.0% | **4.0%** | 33.8 | 29.2% | 37.0% | **0.2%** | 14.3% | 14.3% | 71.4% | **0.1%** | 0.0% | 0.0% | 100.0% |
|  |  | Spring_bud | **3.1%** | 72.9% | 22.7% | 4.4% | **2.8%** | 46.4 | 39.2% | 14.4% | **0.0%** | 0.0% | 0.0% | 0.0% | **0.1%** | 50.0% | 0.0% | 50.0% |
